# Impact of Exercise Therapy on Outcomes in Patients with Low Back Pain: An Umbrella Review of Systematic Reviews

**DOI:** 10.3390/jcm14175942

**Published:** 2025-08-22

**Authors:** Dmitriy Viderman, Sultan Kalikanov, Zhuldyz Myrkhiyeva, Shakhrizat Alisherov, Mukhit Dossov, Serik Seitenov, Yerkin Abdildin

**Affiliations:** 1 Department of Surgery, School of Medicine, Nazarbayev University, Astana 010000, Kazakhstan; 2 Department of Anesthesiology, Intensive Care, and Pain Medicine, National Research Oncology Center, Astana 010000, Kazakhstan; 3 Department of Life Sciences, School of Sciences and Humanities, Nazarbayev University, Astana 010000, Kazakhstan; 4 Department of Electrical Engineering, School of Engineering and Digital Sciences, Nazarbayev University, Astana 010000, Kazakhstan; 5 Department of Anesthesiology, Intensive Care, and Pain Medicine, Medical Center Hospital of the President’s Affairs Administration of the Republic of Kazakhstan, Astana 010000, Kazakhstan; 6 Department of Mechanical and Aerospace Engineering, School of Engineering and Digital Sciences, Nazarbayev University, Astana 010000, Kazakhstan; yerkin.abdildin@nu.edu.kz

**Keywords:** low back pain, exercise therapy, systematic review, pain

## Abstract

**Objective**: This umbrella review aims to analyze the effectiveness of exercise therapy for low back pain through an analysis of systematic reviews that evaluate pain reduction, quality of life improvement, and functional outcomes. **Methods**: This review adhered to PRISMA guidelines and systematic review of review recommendations by searching across PubMed, Scopus, and the Cochrane Library. This study searched for systematic reviews alongside meta-analyses that evaluated exercise interventions in treating low back pain (LBP). This study included reviews that examined exercise therapy for LBP patients and presented data regarding their pain intensity, disability, and quality-of-life outcomes. Data extraction and quality assessment were performed independently by several reviewers. The methodological quality of the included systematic reviews was assessed using the AMSTAR 2 tool. **Results**: This research yielded 88 systematic reviews from 997 evaluated records. Reduction of pain emerged as the primary measured outcome in systematic reviews (81.8%, n = 72), and these studies showed significant improvement rates of 83.0%. The proportion of studies that concluded no change was 9.1%. The most frequently studied exercises were strengthening, aerobic, and mind–body exercises. The reviews reported quality of life improvements in 27.3% (n = 24), but most reviews (68.2%) did not assess this outcome. No studies indicated worsening outcomes. Exercise interventions demonstrated various forms that effectively contribute to LBP management, according to the study results. **Conclusions**: This umbrella review of 88 systematic reviews highlights that exercise therapy is a safe, effective, and commonly used strategy for managing low back pain. However, key limitations include the low methodological quality of several included reviews, risk of bias, imprecision, limited reporting of adverse effects, and confounding from multicomponent interventions. While there is limited certainty that any one type of exercise is more effective than others, individualized approaches and patient adherence appear to be critical factors in optimizing outcomes.

## 1. Introduction

Low back pain (LBP) is one of the leading causes of disability and is defined as “pain in the area on the posterior aspect of the body from the lower margin of the twelfth ribs to the lower gluteal folds with or without pain referred into one or both lower limbs that lasts for at least one day,” according to the study by Global Burden of Disease [1]. It is considered the leading disorder in terms of years of life lost and is ranked sixth for overall disability-adjusted life years, with the global point prevalence being 9.4% [1]. Moreover, LBP was associated with lower physical functioning, higher levels of bodily pain, physical impairment, and depressive symptoms, and lower health-related quality of life [2].

Current recommendations in the management of LBP do not favor surgery, injections, or opioids, which were widely applied in the past. Studies show that combining different approaches, such as patient education, exercise therapy, and behavioral psychotherapy, is recommended [3]. For acute non-specific LBP, initial conservative treatment with a “wait and see” approach is usually advised, since this condition typically has a favorable prognosis and subsides in most patients within 6 weeks. Non-specific acute LBP, on the other hand, is easily managed by simple physiotherapy, whereas chronic non-specific LBP demands a broader approach that integrates both physical and psycho-social interventions [3].

Exercise therapy benefits the musculoskeletal system, reduces pain, improves mood and quality of life, and addresses psychosocial aspects of LBP, making it a versatile management modality [3]. However, the evidence supporting different exercise types remains inconsistent. A variety of approaches, such as aerobic exercise, resistance training, and core stabilization, have been studied, but findings are often contradictory or based on small, isolated trials with varied populations and outcomes. While some reviews suggest the benefits of certain modalities, others report no clear advantage over general exercise. Additionally, methodological heterogeneity, small sample sizes, and differing outcome measures create confusion when seeking the most effective interventions. Prior systematic reviews tend to focus on a single type of exercise, specific subpopulations, or specific timeframes, lacking comprehensive comparison across modalities. Therefore, mapping the most commonly used exercise types, comparing their effectiveness across outcomes like pain, disability, and quality of life, and assessing the quality and consistency of the evidence is warranted [3].

Exercise therapy is now accepted as an essential component of LBP management, given its complexity as being more than a mere relief therapy, but with the potential for enhancing functional mobility, quality of life, and preventing further recurrences. Knowing the impact of various forms of exercises applied to those patients, including aerobic activities, stabilization, and strengthening exercises, can help in developing targeted therapy programs and improving overall results. Furthermore, evaluating the impact of exercise therapy allows identification of optimal long-lasting interventions, potentially avoiding surgeries and pharmacological management, thus improving cost-effectiveness in LBP care [1,2,3].

The objective of this umbrella review is to systematically synthesize, map, and evaluate evidence from existing systematic reviews and meta-analyses on physical exercise therapy for low back pain. This includes identifying the most frequently studied types of exercises, assessing their comparative effectiveness, mapping reported outcomes, and analyzing the magnitude of effect and methodological quality by exercise type.

## 2. Materials and Methods

We followed the PRISMA (“Preferred Reporting Items for Systematic Reviews and Meta-Analyses”) [4] guidelines for the conduct of this umbrella review, supplemented by recommendations for conducting systematic reviews of systematic reviews [5]. The protocol was registered in the OSF Registry (https://osf.io/registries/drafts/6867aebdafb60d4c510463f4/review, accessed on 14 August 2025).

### 2.1. Inclusion Criteria

Study types: our inclusion criteria included systematic reviews, with or without meta-analysis;

Patient population: patients suffering from LBP;

Intervention: any type of exercise used in the management of LBP;

Comparison: placebo, alternative treatment, or no treatment;

Outcomes: improvement in LBP, quality of life.

The timeframe of outcomes measurement can be short-term, intermediate-term, and long-term. Short-term outcomes are defined as assessments conducted less than 3 months post-intervention, intermediate-term as between 3 and 12 months, and long-term as more than 12 months’ follow-up.

### 2.2. Exclusion Criteria

Studies that did not meet the inclusion criteria were excluded. Studies that are not systematic reviews or meta-analyses, such as randomized controlled trials, observational studies, and case reports, were excluded. In addition, studies that focus on invasive interventions (e.g., nerve blocks, injections, implants) or surgical procedures were excluded. We also excluded studies written in languages other than English due to a lack of resources for translation and access to multilingual screening tools.

Within this umbrella review, we systematically mapped the different types of exercises that have been used in the management of low back pain.

The following items were covered: the timeline of published systematic reviews; classification of exercise therapy used for LBP as physical activity programs designed to improve strength, flexibility, mobility, or overall physical function, delivered with therapeutic intent (with exclusion of reviews primarily focused on surgical procedures, pharmacological treatments, or invasive techniques); categories of outcomes of exercise therapy used for LBP; and the quality-of-life outcomes after exercise therapy used for low back pain management.

### 2.3. Database Search

We developed a comprehensive search strategy using PubMed, Scopus, and the Cochrane Library. We used the following search terms and their combinations:

(“low back pain”[MeSH Terms] OR “low back pain”[All Fields] OR “lumbago”[All Fields])

AND

(“exercise”[MeSH Terms] OR “exercise therapy”[MeSH Terms] OR “physical activity”[All Fields] OR “resistance training”[All Fields] OR “aerobic”[All Fields] OR “yoga”[All Fields] OR “Pilates”[All Fields])

AND

(“systematic review”[Publication Type] OR “meta-analysis”[Publication Type])

The search strategy included terms related to low back pain and exercise interventions. The search was conducted on 27 September 2023, and included all eligible articles from database inception to that date. Appropriate indexing terms and Boolean operators were adapted for each database. There were no date restrictions applied, and only studies published in English were included.

All titles and articles that met the inclusion criteria were downloaded in full text and checked by several independent reviewers. The reasons for exclusion were noted. We also scrutinized the references of the included articles for additional relevant publications.

### 2.4. Data Extraction

Sata were extracted from the included SRs and independently checked by the reviewers. If the data were in doubt, the original articles were checked. For the narrative analysis, we extracted detailed characteristics, such as authors’ names, diagnosis, number of patients, type of physical exercise, pain reduction and possible mechanisms, and study conclusions. Two reviewers independently screened titles and abstracts. Next, full-text screening of potentially eligible articles was performed. Disagreements were resolved through discussion by consulting a third reviewer.

### 2.5. Quality Assessment

We used the AMSTAR 2 tool, A MeaSurement Tool to Assess systematic Reviews, to determine the methodological quality of the systematic reviews included in the study. AMSTAR 2 is an exhaustive and reliable tool for assessing the quality of SRs with good construct validity [6]. It consists of 16 items, of which 7 items are noted as essential indices for measuring the reliability of the review. Every item of the assessment is categorized as a “Yes,” “No,” or “Partially Yes” depending on the presence of the criterion in the assessment.

### 2.6. Data Synthesis

Meta-analysis was not performed in this umbrella review due to several reasons:

- heterogeneity in populations;
- heterogeneity in interventions;
- heterogeneity in outcomes;
- heterogeneity in comparators;
- potential overlap of primary studies.

To summarize the evidence, a narrative synthesis was conducted.

## 3. Results

### 3.1. Search Results and Included Studies

**Figure 1:**
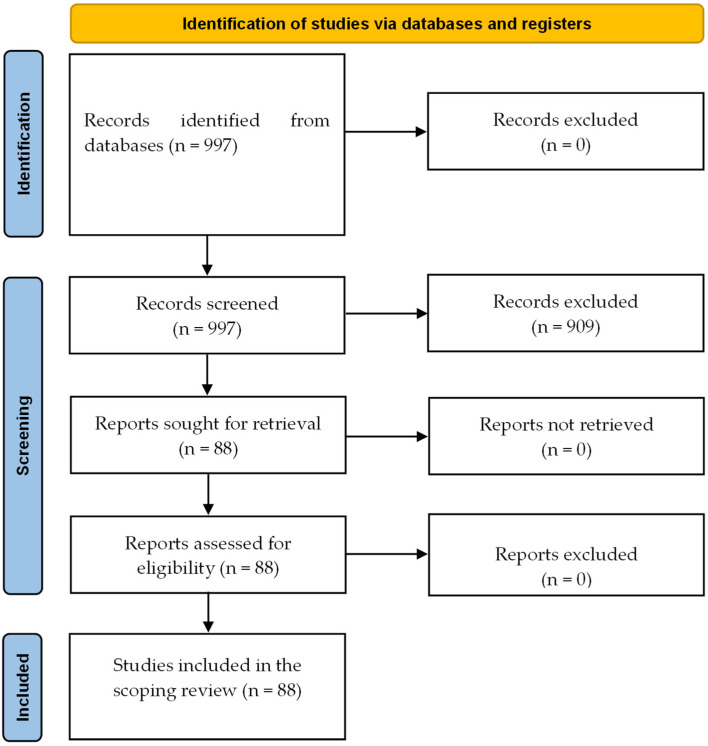
PRISMA diagram.

We initially identified 997 publications. After removal of duplicates, abstract screening, and assessment of full-text articles, 88 systematic reviews met the inclusion criteria and were included in this umbrella review (Figure 1, Table 1).

### 3.2. Characteristics of Publications

**Figure 2:**
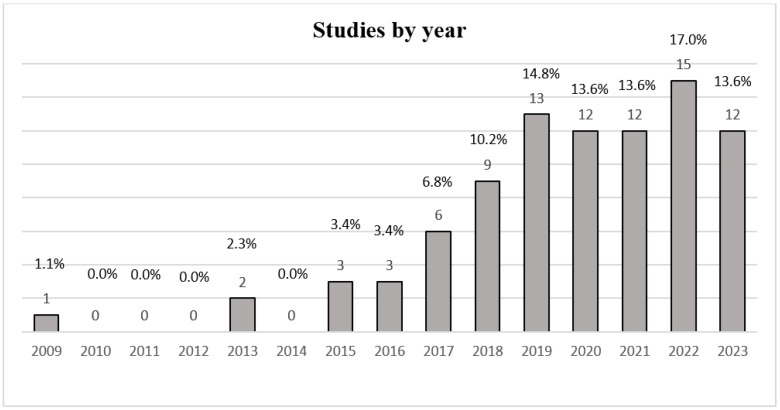
The publication timeline and the annual percentage and count of studies conducted for the period from 2009 to 2023.

Figure 2 presents a bar graph illustrating the annual percentages of research studies across the years 2009 to 2023. The number of studies shows a moderate increase over time, reaching its highest point in 2022 at 17%; this corresponds to 15 studies [10,17,19,20,21,22,23,24,25,26,28,29,30,31,37]. In contrast, in 2009, the lowest percentage of studies was recorded at 1.1%, equivalent to one study [74]. It should be noted that no studies were reported for the years 2010, 2011, 2012, and 2014.

### 3.3. Classification of Exercise Therapy for LBP

#### Characteristics of Exercises

**Figure 3:**
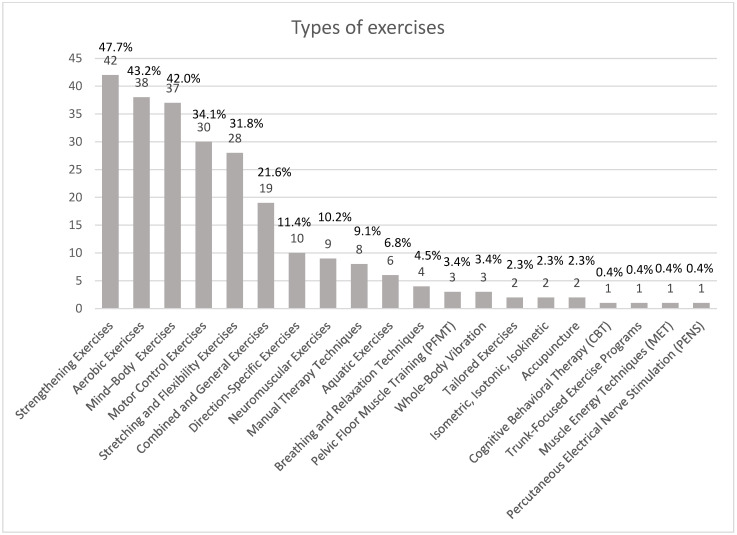
Distribution of exercise types used for non-specific low back pain.

Strengthening exercises were the most commonly reported, followed by aerobic and mind–body exercises. Motor control and flexibility exercises, combined and general exercises, and direction-specific exercises also play significant roles. Neuromuscular exercises, manual therapy techniques, and aquatic exercises are moderately used. Less common methods include breathing and relaxation techniques, pelvic floor muscle training (PFMT), whole-body vibration therapy, tailored exercise, isokinetic therapy, isotonic therapy, acupuncture, cognitive behavioral therapy (CBT), trunk-focused exercise programs, muscle energy technique (MET), and percutaneous electrical nerve stimulation (PENS), each accounting for a minimal percentage of usage in therapy programs. This diversity underscores the tailored approach needed in physical therapy for low back pain. Figure 3 shows the distribution of different exercise types used in physical therapy for chronic non-specific low back pain. The bar chart ranks the types of exercises by their prevalence in therapy programs.

**Figure 4:**
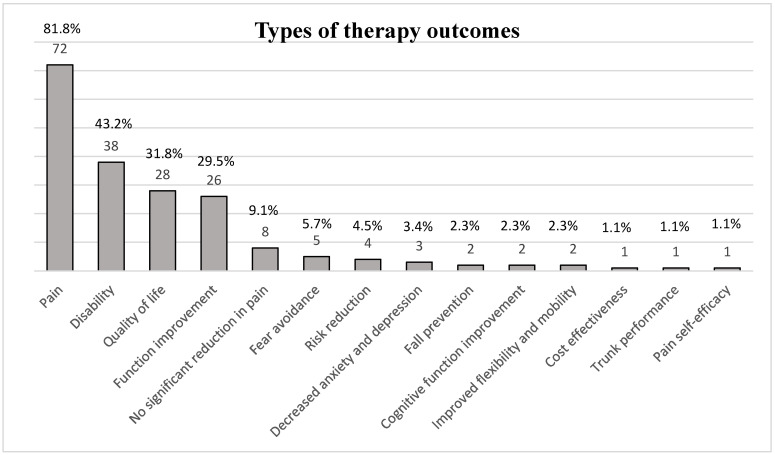
Categories of exercise therapy outcomes. Bar chart of the prevalence of therapy outcomes in the management of low back pain management.

Eighty-eight publications on exercise therapy for low back pain management were included, revealing the variety and frequency of exercises used. Figure 4 shows a breakdown of the results.

Strengthening exercises were the most commonly reported exercise type in the publications, as shown by 42 out of 88 studies [7,9,13,17,18,20,24,25,28,29,32,34,35,36,41,42,45,49,50,51,52,55,57,59,66,67,70,71,76,77,78,82,83,85,86,87,88,89,91,92,93,94]. This group includes a variety of exercises such as wall push-ups, endurance training, and mild weightlifting. Strengthening exercises are popular, which indicates that there is general agreement on their value in improving muscular strength and stability in the treatment of low back pain. Aerobic workouts come in as a close second, according to 38 studies [7,9,20,22,24,28,29,31,32,34,35,36,40,41,42,43,45,46,51,52,55,56,57,58,59,67,70,71,75,76,77,78,82,83,87,88,91,92]. This includes exercises like walking, swimming, cycling, gymnastics, and other similar activities. These are essential for both pain management and recuperation. Thirty-seven publications [7,8,9,10,12,16,17,22,24,29,33,34,35,36,40,41,42,43,44,45,46,47,48,52,55,56,57,61,65,66,67,68,77,82,84,89,90] describe various mind–body practices, including yoga, qigong, tai chi, Pilates, and baduanjin. The management of low back pain can be greatly aided by these exercises because of their acknowledged significance in improving stress reduction, flexibility, and mind–body awareness. In 30 studies [10,12,20,22,23,26,27,28,29,33,35,36,37,43,44,45,52,54,56,57,71,72,77,78,83,86,89,90,93,94] motor control activities are included, indicating that core, balance, and stability exercises are recognized as effective treatments for low back pain. There are 28 publications [7,8,9,17,20,24,25,28,32,34,35,40,45,55,56,57,64,71,74,76,77,82,86,87,88,91,93,94] that describe the benefits of stretching and flexibility exercises, highlighting how they can increase range of motion and decrease muscular tension. Nineteen studies [10,12,14,21,24,34,45,49,53,57,58,59,71,76,78,81,85,86,93] report on different combinations of workouts or general fitness regimens that emphasize a multimodal, holistic approach to rehabilitation. Ten studies [10,12,22,24,34,35,41,55,57,73] mention exercises that focus on movements in certain directions, such as McKenzie exercises, indicating a more focused approach to treating low back pain.

The less frequently reported types of exercises, such as neuromuscular exercises, manual therapy techniques, aquatic exercises, etc., highlight a broad spectrum of interventions available for clinicians, though these may be more specialized or used as complementary therapies. This variability highlights the complexity of treating low back pain and the necessity for tailored treatment plans that take into account the requirements, preferences, and unique features of each patient, as well as their disease.

### 3.4. Characteristics of Outcomes

The vast majority of studies (81.8%; 72 studies) [7,8,9,10,11,12,13,14,15,16,17,18,19,22,23,24,26,27,29,30,34,35,36,37,38,39,40,41,42,43,44,46,47,49,50,52,53,54,57,58,59,60,61,62,63,64,65,66,67,68,70,72,73,74,75,76,77,78,79,80,81,82,84,85,86,87,89,90,91,92,93,94] focused on the role of exercise in pain reduction. This highlights the crucial role that pain reduction plays in the treatment and evaluation of low back pain management programs. Reduction of disability [7,8,10,11,14,15,16,22,23,24,26,27,28,29,30,31,32,39,42,44,47,51,54,61,66,69,70,72,73,74,75,76,80,83,86,91,92,93], improvement of quality of life [7,8,9,11,14,15,16,17,18,23,24,28,36,37,38,40,41,44,47,54,58,59,70,71,72,76,82,93], and function improvement [12,13,20,21,22,23,24,27,28,31,33,34,35,36,37,38,42,43,55,57,62,66,69,79,82,85] are also often reported outcomes (43.2%, 31.8% and 29.5%, accounting for 38, 28, and 26 studies, respectively), suggesting a strong emphasis on improving patients’ physical capacity and quality of life. Other outcomes, such as fear avoidance, risk reduction, and fall prevention, are less commonly reported but significant, since they address the psychological and safety issues linked with low back pain. While less common in the research, outcomes such as reduced anxiety and depression, enhanced cognitive function, and increased flexibility and mobility hint at a more holistic approach to therapy that takes into account the broader effects of low back pain on patients’ entire well-being. Figure 4 provides a concise overview of the main outcomes of 88 SRs on the use of exercise therapy to treat lower back pain.

### 3.5. The Impact of Exercise on Pain Outcomes

**Figure 5:**
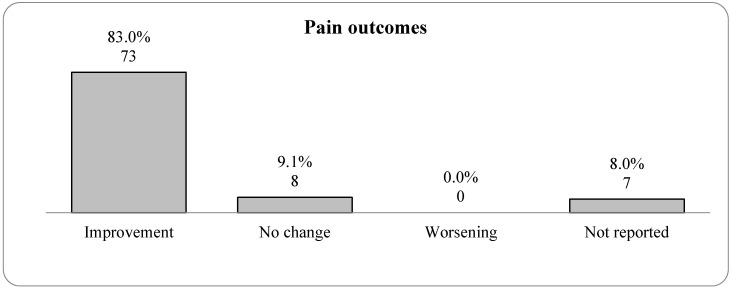
Bar graph representing the distribution of pain outcomes after therapies were applied for low back pain.

A significant majority of the studies (83.0%; 73 studies) [7,8,9,10,11,12,13,15,16,17,18,19,21,22,23,24,26,28,29,30,31,32,34,35,36,37,39,40,42,43,44,46,47,49,50,51,52,53,54,55,56,57,60,61,62,63,64,66,67,68,70,71,72,73,74,75,77,78,79,80,81,82,84,85,86,87,89,91,92,93,94] indicated an improvement in pain levels among participants, whereas 9.1% (8 studies) [10,14,20,27,38,41,65,90] found no difference in pain reduction. Pain was commonly assessed using a Visual Analog Scale (VAS) or Numeric Rating Scale (NRS), though not all studies specified the tool. Variability in measurement may have limited comparability across reviews. Notably, none of the investigations found that the discomfort worsened. In addition, pain outcomes were not reported in 8.0% (seven studies) [25,33,45,48,69,83,88]. This figure supplements the data in Figure 5, which classifies pain self-efficacy as a separate outcome category and lists 72 studies that indicate decreased pain. However, this figure groups outcomes linked to pain self-efficacy under the “Improvement” category, seeing any reduction in pain as an improvement. Pain self-efficacy is important in pain management because it influences a patient’s confidence in their capacity to control their pain, resulting in more effective management behaviors, better coping techniques, and perhaps improved pain perception, decreasing both real and perceived pain levels. The inclusion of this psychological feature in the “Improvement” category is acceptable, emphasizing the constant focus across research on the favorable effects of therapies on low back pain. Figure 5 presents a bar graph depicting the distribution of pain outcomes from a series of studies focusing on low back pain therapies.

### 3.6. The Impact of Exercise on Quality of Life

**Figure 6:**
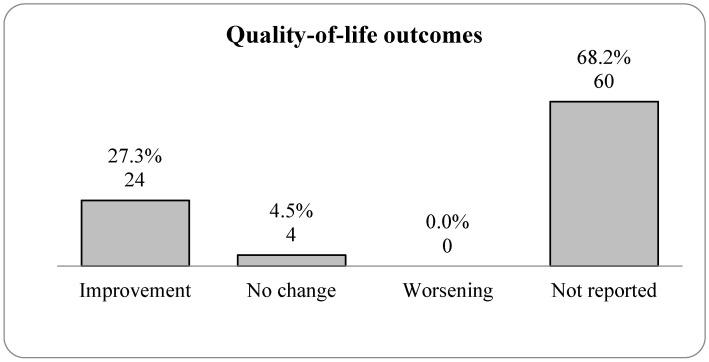
Bar graph illustrating quality-of-life outcomes after various therapies were applied for low back pain.

The therapies contributing to these outcomes include strengthening exercises like light weightlifting, aerobic exercises such as walking and swimming, and mind–body practices including yoga and tai chi, as shown in Figure 4. These exercises were integral to reducing pain and disability, as shown in Figure 5, which significantly contributed to the improved quality of life reported by 27.3% of participants (24 cases) [7,8,9,11,13,15,16,17,18,23,24,28,36,37,39,40,44,54,58,70,71,72,82,93]. Additionally, 4.5% (four cases) [38,41,47,76] experienced no change in their quality of life, and no worsening conditions were reported (0.0%). A notable 68.2% (60 cases) [10,12,14,19,20,21,22,25,26,27,29,30,31,32,33,34,35,42,43,45,46,48,49,50,51,52,53,55,56,57,59,60,61,62,63,64,65,66,67,68,69,73,74,75,77,78,79,80,81,83,84,85,86,87,88,89,90,91,92,94] of the outcomes were not detailed, aligning with the study’s primary focus on pain outcomes. Figure 6 illustrates the impact on quality of life following the application of different therapeutic exercises for managing various types of low back pain.

### 3.7. Quality Assessment Results

The methodological quality of the included publications, assessed via AMSTAR 2, presented in Table 2, revealed that over 50% (47/88) were critically low quality [14,16,17,18,20,21,23,26,27,28,29,30,31,32,33,38,41,44,45,46,48,50,51,53,55,56,57,58,59,61,65,66,67,68,70,74,83,85,86,87,88,89,90,91,92,93,94], while 30 publications were low quality [7,8,9,11,12,13,24,25,35,36,39,42,43,47,49,52,54,60,62,63,64,73,75,76,77,78,79,80,81,82]. Most of the studies (n = 54) lacked reporting on item 7 (“Did the review authors provide a list of excluded studies and justify the exclusions?”), which could lead to the unreasonable exclusion of potentially relevant studies, thereby reducing the credibility of the evidence. Moreover, 44 studies failed to assess publication bias (item 15: “If they performed quantitative synthesis did the review authors carry out adequate investigation of publication bias (small study bias) and discuss its likely impact on the results of the review?”), which could significantly distort the results of the systematic reviews and increase the risk of overestimation of the effectiveness of exercise therapy on the management of low back pain, which in turn could lead to biased decision-making by healthcare professionals. Only three publications had moderate methodological quality [10,19,22]. High quality was observed in eight studies [15,34,37,40,69,71,72,84]. The publications with high quality had no flaws or only one non-critical flaw regarding the reporting of funding (item 10) or conflict of interest (item 16), which are frequently overlooked items regarding the transparency of results. The lack of reporting on these items prevents the assessment of the impact of commercial interests or any other conflicts on adequate interpretation of findings.

AMSTAR questions (Qs)

1. Did the research questions and inclusion criteria for the review include the components of PICO?

2. Did the report of the review contain an explicit statement that the review methods were established prior to the conduct of the review, and did the report justify any significant deviations from the protocol?

3. Did the review authors explain their selection of the study designs for inclusion in the review?

4. Did the review authors use a comprehensive literature search strategy?

5. Did the review authors perform study selection in duplicate?

6. Did the review authors perform data extraction in duplicate?

7. Did the review authors provide a list of excluded studies and justify the exclusions?

8. Did the review authors describe the included studies in adequate detail?

9. Did the review authors use a satisfactory technique for assessing the risk of bias (RoB) in the individual studies that were included in the review?

10. Did the review authors report on the sources of funding for the studies included in the review?

11. If meta-analysis was performed, did the review authors use appropriate methods for statistical combination of results?

12. If meta-analysis was performed, did the review authors assess the potential impact of RoB in individual studies on the results of the meta-analysis or other evidence synthesis?

13. Did the review authors account for RoB in individual studies when interpreting/discussing the results of the review?

14. Did the review authors provide a satisfactory explanation for, and discussion of, any heterogeneity observed in the results of the review? 

15. If they performed quantitative synthesis, did the review authors carry out adequate investigation of publication bias (small study bias) and discuss its likely impact on the results of the review?

16. Did the review authors report any potential sources of conflict of interest, including any funding they received for conducting the review?

### 3.8. Magnitude of Effect and Study Quality by Exercise Type

**Figure 7:**
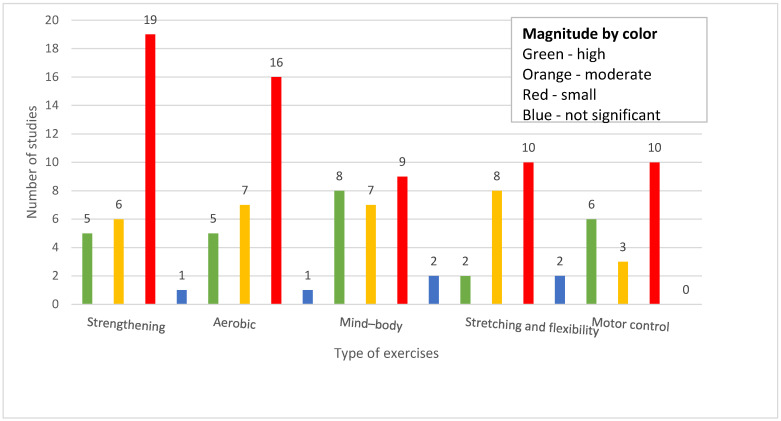
Grouped bar chart illustrating the magnitude of effect size per exercise type.

**Figure 8:**
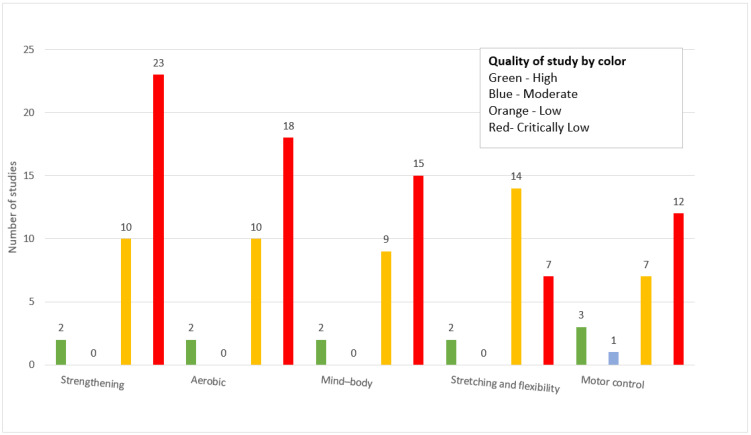
The quality of the included studies per exercise type.

Table 3 summarizes the effectiveness of exercise by type on low back pain and the studies’ methodological quality. Figure 7 depicts how exercise interventions for low back pain are associated with varying effect sizes in studies. Among 73 studies from Table 3, 2 studies did not conduct a meta-analysis [82,94]. Both systematic reviews without meta-analyses concluded that there was limited evidence to support the use of exercise for pain reduction. One study analyzed cost-effectiveness and concluded that exercise therapy was cost-effective in comparison to usual care [71]. Since these three studies did not report effect size, they were not included in Figure 7. Strengthening, aerobic, and mind–body exercises had the greatest number of studies reporting high pain reduction (five studies each). However, most studies across all exercise types reported small or moderate effects. Performance-oriented exercises had the fewest reports of high or moderate effectiveness and the highest proportion of nonsignificant results. Effect size was presented heterogeneously in forms of mean difference, standardized mean difference (SMD), odds ratio (OR), risk ratio (RR), Hedges’ g value, and weighted mean difference (WMD). Effect sizes were categorized as low, moderate, and high based on established thresholds. For standardized metrics (SMD and Hedges’ g), low was defined as 0.2–0.49, moderate as 0.5–0.79, and high as more than 0.8. For ORs and RRs, values of 1.01–1.49 were considered low, 1.50–1.99 moderate, and more than 2.00 high. Mean differences and WMDs were interpreted using clinical judgment, typically defining a more than 2-point change on a 10-point scale as high. Figure 8 displays the methodological quality of studies by exercise type based on AMSTAR 2. The highest proportion of studies across all categories was low or critically low quality, specifically those evaluating strengthening and aerobic exercises, which had 23 and 17 critically low-quality studies, respectively. Only a small number of studies were rated as high-quality—notably, strengthening, aerobic, and mind–body exercises had two high-quality studies each.

A total of 14 studies included direct comparisons of two or more exercise types. Across these, the following relative patterns of effectiveness for pain reduction were identified:

- Pilates was more effective than general exercise and direction-specific exercise, with low certainty of evidence [10].
- An outcome matched to exercise treatment may be more effective than an unmatched primary outcome [52].
- Pilates, mind–body exercise, and core-based exercise were the most effective interventions [24].
- Motor control is only effective in combination with musculoskeletal therapies [27].
- Pilates and McKenzie therapies are better than flexibility, minimal treatment, and other effective exercises [35].
- Low back exercise in combination with health education might be the best approach [40].
- It is not clear whether a region-specific or general-specific approach is better for pain reduction [41].
- Walking is better than yoga in the short term, and yoga is better than walking in the intermediate term [46].
- Multidisciplinary-based rehabilitation may be better than active physical intervention [49].
- Primary outcome-matched exercises may be better than the unmatched category [52].
- Baduanjin exercise is more effective than general exercise or general exercise with routine drugs (ibuprofen) [61].
- Stabilization exercise is better than general exercise [86].
- Isometric and motor control exercises are both effective, and motor control exercises are more effective than isometric exercises [26].
- Individualized exercise is better than active exercises [22].
- Regarding insignificance and possible reasons for it:
- Two studies reported high risk of bias and heterogeneous interventions [14,66].
- Three studies reported a small sample size [14,63,66].
- One study analyzed a specific population of older adults [65].
- One study was comparative and found equivalent effectiveness between walking and other physical exercises [76].
- One study investigated incidental physical activity as a planned program [63].
- Regarding cost-effectiveness:
- One review assessed cost-effectiveness and found that exercise therapy was more cost-effective than usual care for subacute and chronic low back pain, but was not superior to other active treatments. From a healthcare perspective, exercise therapy demonstrated favorable cost-utility ratios (CURs), ranging from GBP 3760 to GBP 17,447 per quality-adjusted life year (QALY) gained, indicating cost-effectiveness. However, from a societal perspective, the findings were mixed; one analysis reported a high CUR of GBP 82,657 per QALY gained, suggesting that exercise therapy may not be cost-effective compared to usual care in broader economic models [71].

## 4. Discussion

### 4.1. Summary of Key Findings

The objective of this review was to systematically map and discuss the different types of exercises that have been used for low back pain. We identified that, among the exercise types, the most commonly used exercise was strengthening exercise, accounting for 42 out of the 88 included studies (47.7%). The least used exercise was mind–body exercise such as yoga or tai chi, accounting for 37 out of the 88 included studies (42.0%), and aerobic exercise accounted for 38 out of the 88 included studies (43.2%). Other types of exercises were motor control, stretching exercises, and direction-specific interventions. Infrequent forms of intervention included individual treatment techniques such as manual therapy, exercises performed in water, and breathing exercises. The clinical outcomes mainly concerned decreasing pain, which was mentioned in 72 out of 88 studies (81.8%). Increasing quality of life was less often described, being observed in 24 studies (27.3%). This likely reflects a long-standing focus on pain intensity as the primary outcome in low back pain research, despite growing recognition of the importance of biopsychosocial outcomes such as well-being. Studies may have lacked the resources or study duration needed to capture longer-term impacts on quality of life. Disability reduction and functional change were two other outcomes reported by 43.2% and 29.5% of the studies, respectively. Most importantly, none of the studies reported worsening symptoms, and a small percentage of them (9.1%) found no change. This may be explained by methodological limitations such as high risk of bias, small sample sizes, and heterogeneous interventions. In some cases, equivalent effectiveness between interventions or the focus on specific populations may have reduced the ability to detect clear benefits. These factors likely contributed to inconclusive or minimal effects observed in certain reviews. In general, the results indicate that decreasing pain levels continues to be a focus of the research. Pain was commonly assessed using VAS or NRS, though not all studies specified the tool. Variability in measurement may have limited comparability across reviews. Further, strengthening exercises are still the most commonly used type of exercise for LBP management, ahead of aerobic and mind–body exercises. The findings from these studies imply that a personalized, biosocial intervention based on the complexity of aerobic and resistance training may be very helpful in the treatment of LBP. Pilates, mind–body exercises, core-based training, and individualized approaches showed more favorable outcomes in direct comparisons. These findings are aligned with recent network meta-analyses and suggest that targeted or tailored exercise modalities may be more effective than generalized programs for pain reduction in low back pain. Differences in results across the included systematic reviews may be partly due to substantial heterogeneity in population characteristics, intervention protocols, and methodological quality. Few reviews focused on specific subgroups, such as older adults or individuals engaged in incidental physical activity, which may limit the generalizability of their findings to the broader population with chronic low back pain. Differences in duration, frequency, and intensity of exercise further complicate direct comparisons among studies. Furthermore, the included reviews reported effect sizes using a variety of statistical measures, making it difficult to synthesize and interpret results across studies.

### 4.2. Comparison with the Existing Literature

Our results are partly consistent with the results in the existing literature. For example, a study by Saragiotto et al. (N = 29, n = 2431) aimed to evaluate the effectiveness of motor control exercise (MCE) in the management of chronic non-specific low back pain [95]. It was found that MCE affected short-term, intermediate-, and long-term pain improvement compared to minimal intervention. However, no evidence was found supporting MCE for pain management, compared to other types of exercises. This implies that exercises in general are effective in pain management. Similarly, Gianola et al. (N = 4, n = 8765), who studied the effectiveness of different interventions for the management of acute and subacute non-specific low back pain, found that exercise was among the most efficacious treatments (SMD: −1.4, 95%CI: −2.41 to −0.40), along with heat wrap, opioids, and manual therapy [96]. In contrast, a study by IJzelenberg et al. (N = 23, n = 2674) provided low-certainty evidence that exercise therapy does not cause clinically important differences in acute non-specific low back pain and functioning in the short term compared to sham or placebo treatment [97]. One quasi-randomized trial of 299 patients reported a small difference in pain scores between the exercise and placebo group (mean difference—0.80) and no significant change in functional status. When comparing exercise therapy with no treatment, two studies produced inconsistent results: one observed minimal improvement, and another displayed no variation.

### 4.3. Study Strengths and Limitations

One of the strengths of this study is that it presents a synthesis of exercise therapy efficacy for LBP based on 88 studies and 2674 participants. This highlights the broad range of exercises common in the treatment process, including strengthening exercises, aerobic exercises, and mind–body exercises, which makes the strategies applied to patients more generalized and thus suited to the individual patient’s needs. In addition, this research focuses on outcomes, such as pain relief, changes in quality of life, and functions, which help make the present work valuable for clinicians. By dividing LBP into more refined subcategories and having a separate classification for exercise interventions, as well as having greater use of bar charts and figures for year-by-year comparisons, it is possible to see key trends and results regarding the efficacy of exercise therapy year by year.

However, the results from this study have several weaknesses originating from low certainty of evidence resulting from the risk of bias and imprecision from small sample sizes and methodological shortcomings such as lack of blinding. Some evidence suggests that patients with low back pain may benefit more from certain types of exercise. For example, exercises that integrate the mind and body, such as yoga [46,47] and qigong [19], are particularly helpful for chronic low back pain. This is likely due to their emphasis on relaxation, flexibility, and fear reduction. In contrast, motor control exercises may be more effective for recurrent low back pain, where there is impaired trunk stability or movement patterns [77,93]. However, only a few reviews have stratified their results by acute and chronic status, making it difficult to draw definitive conclusions. This highlights the need for future systematic reviews and primary studies to conduct more detailed subgroup analyses to personalize treatment. This umbrella review included systematic reviews of varying methodological quality, including some rated as low or critically low using AMSTAR 2. While this decision allowed for broader inclusion, it may have diluted the strength of the conclusions. Furthermore, fewer studies compared the findings regarding exercise therapy with no treatment at all, and even fewer reports highlighted adverse effects to ensure total comprehension of the safety of exercise therapy. Likewise, several included reviews examined multicomponent interventions, where exercise was combined with treatments such as cognitive-behavioral therapy, education, or manual therapy. This heterogeneity introduces potential confounding, making it difficult to attribute outcomes solely to exercise therapy. Another major limitation is that the point of interest, apart from pain relief, is often mentioned vaguely or not at all, which means that outcomes such as quality of life and mental state were not assessed in many trials, or were assessed inadequately. This absence of a broad outcome profile limits the potential to draw definite conclusions regarding the efficacy of exercise therapy for low back pain. Finally, limiting the review to English-language studies may have introduced language bias.

### 4.4. Implications for Practice and Policy

The results from this study should be used to inform the development of policies that incorporate exercise therapy as a fundamental part of low back pain treatment. A comprehensive pattern of intervention should include networking of different types of exercise, including strength, aerobic, and mind–body exercises, to personalize care under general treatment norms. Existing structured models, such as the STarT Back stratified care approach in the UK [98] and the BetterBack MOC program in Sweden [99], are examples of scalable, evidence-based frameworks that integrate physical and psychosocial components and could inform future education and clinical practice. The adoption of a standardized outcome framework, such as the WHO International Classification of Functioning, Disability and Health (ICF) model [100], may improve consistency in outcome reporting across studies of low back pain. This will allow comparison of different interventions by combining measures of pain, function, and quality of life within a single framework. Targeted programs that aim at making structured exercise interventions available to patients both from clinical and community environments should be financially embraced by policymakers, as the results from this study showed improvements in the areas of pain mitigation and quality of life. In addition, policies should promote the development of educational programs for instructors in delivering multiple exercise therapies to patients and ensuring that patients have equal access to these treatments, particularly for patients in underfunded physical therapy service areas.

### 4.5. Implications for Further Research

**Figure 9:**
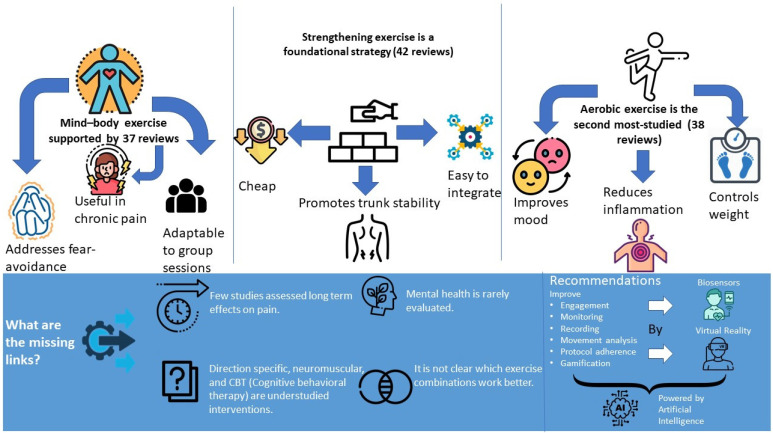
Summary of key findings, research gaps, and future directions in exercise therapy for low back pain.

Finally, further studies should be conducted to fill the gaps in the evidence found in this study, especially the effects of exercise therapy in the long run on pain and other domains, such as quality of life, psychological health, and functioning level. Future investigations of exercise therapy that consist of larger and more diverse subjects with more strict and detailed protocols are required to increase the conclusiveness of the studies and examine the safety of exercise therapy for various clients. Considering that quality of life and mental health outcomes were reported less frequently than pain-related measures, which likely reflects a traditional focus on pain intensity rather than broader biopsychosocial impacts, future research should address this gap by including validated measures of well-being and psychological health. The quality of more than half of the included systematic reviews was rated as “critically low” by AMSTAR 2, limiting the reliability of the findings of this comprehensive review. Including these studies provided a broader overview but increased the risk of bias and imprecision. As a result, the overall certainty of the evidence remains low, highlighting the need for higher-quality future reviews. Novel technologies such as virtual reality (VR) and biosensors powered by artificial intelligence and machine learning can be used to improve patient engagement, monitoring, recoding, movement analysis, gamification, and protocol adherence. For instance, a recent systematic review and meta-analysis of 14 randomized trials found that VR significantly reduced pain intensity in chronic spinal pain conditions, including low back pain [101]. Smaller-scale studies using VR-supported exercise platforms like Xbox Kinect-based training have also shown significant improvements in pain, disability, fall risk, and quality of life among older adults with chronic LBP [102]. Exercise interventions should also be investigated that have not been traditionally used for the treatment of LBP, and the best synergy of therapies for certain LBP subtypes should be explored. Priority areas, gaps, and recommendations are summarized in Figure 9. Figure 9 summarizes the key characteristics, benefits, and frequency of use of the three most commonly studied exercise types for low back pain—strengthening, aerobic, and mind–body exercises—while also highlighting research gaps (e.g., limited attention to long-term outcomes and mental health) and recommending future directions such as AI- and VR-supported interventions.

## 5. Conclusions

In summary, an extensive overview of 88 studies of exercise therapy for the management of low back pain has produced a comprehensive list of the most frequently used exercises. Physical exercise appears to be an effective, safe, and affordable method, not only for low back pain prevention and management, but also for many other cardiovascular, respiratory, metabolic, musculoskeletal, and neurological conditions. While many studies report small to moderate pain reduction across most exercise types, some specific forms—such as Pilates, mind–body exercises, and core-based training—tend to show relatively higher effectiveness in comparative analyses.

Research trends over time show a moderate increase in the number of studies conducted from 2009 to 2023, peaking in 2022 with 15 studies (17% of the total). This umbrella review found a large rise in the number of studies on exercise for low back pain, especially in the recent decade. This trend indicates that there is rising acknowledgment of the value of non-pharmacological therapies and the efficacy of exercise. The various exercises used in managing LBP were strengthening exercises, which are the most prevalent. Aerobic and mind–body exercises were also significantly represented, alongside motor control and flexibility exercises, emphasizing a multidisciplinary approach to therapy. Aerobic exercises are closely associated with improved cardiovascular fitness and may have a role in pain management and functional rehabilitation. There were also many SRs focusing on mind–body exercises, which are part of an integrated strategy that treats the psychological elements of chronic pain with physical therapy. Exercises involving motor control and flexibility serve to highlight the treatment goal of improving the body’s stability and range of motion.

Direct comparisons between exercise types suggest that some exercises may outperform others in specific contexts. For instance, Pilates was often more effective than general or flexibility-based programs, and interventions that were individualized or matched to specific outcomes tended to show better results than generic protocols. These findings highlight the importance of tailoring exercise interventions to the needs, preferences, and clinical profiles of patients. Furthermore, adherence appears to play a critical role in treatment effectiveness, emphasizing that the most suitable exercise is often the one the patient is most likely to continue consistently. Nevertheless, the certainty of this evidence remains limited, and the superiority of one approach over another is not consistently demonstrated across all reviews.

The distribution of pain outcomes figure displays the results of pain management, with 83% of the studies showing improvement and 9.1% showing no change. Notably, no studies reported worsening pain. Pain self-efficacy, grouped under “Improvement,” indicates a reduction in pain as a significant therapeutic outcome. Following various therapies, 27.3% reported improved quality of life, with no worsening conditions noted. The majority of outcomes (68.2%) were not detailed, reflecting the primary focus on pain outcomes in the studies. According to the research, there is a strong, multidisciplinary approach to treating low back pain. A range of activities is planned to improve patient outcomes in several physical health domains, with a focus on pain management and functional rehabilitation, as well as a growing appreciation of the importance of psychological and cognitive factors in the healing process.

## Figures and Tables

**Table 1:** Study characteristics.

Author, Year	Study Design	Disease/Pathology, Comorbidities	N of Patients	Total Number of Studies	Type of Physical Exercise (Resistance/Aerobic, Tai Chi)	Pain Reduction Due to Exercise (+/−)	Possible Mechanisms of Pain Reduction/Exercise-Induced Hypoalgesia	Study Conclusions/Comments
Zhang et al., 2023 [7]	SR and MA of RCTs	CLBP	989	18	Comprehensive, strength, mind-body, traditional physical, aerobic, stretching exercises	+	Strengthening muscles, endorphin release, physical and psychological benefits, improved mobility and function, improved muscle function.	Exercise therapy effectively improves pain, dysfunction, and quality of life. Not approved for mobility.
Ferraz et al., 2023 [8]	SR and MA of RCTs	LBP during pregnancy	65	2	Comprehensive exercise, aerobic exercise, mind–body exercise, strength exercise, stretching exercise	+	Pilates may reduce pain through improved muscular strength and stabilization of the lumbar and pelvic regions, which are typically stressed during pregnancy. Enhanced physical stability could lead to decreased strain and discomfort.	Pilates exercise may reduce pregnancy-related low back pain more effectively than usual prenatal care or no exercise. It was recommended that Pilates should be considered a safe and beneficial option for pain management during pregnancy.
Gilanyi et al., 2023 [9]	SR and MA of RCTs	CNSLBP	1121	17	yoga, aerobic exercise, resistance training, combined aerobic and flexibility exercises	+	Higher PSE (pain self-efficacy) is associated with better treatment adherence and improvements in pain, disability, and emotional distress following treatment.	Interventions could be more effective if specifically designed to target key aspects of self-efficacy, potentially leading to better outcomes in pain management and functional abilities.
Wong et al., 2023 [10]	SR and MA of RCTs	CNSLBP	369 for pain + 418 for disability = 787	11	Pilates	+	Pilates may enhance core stability and postural alignment, which can reduce stress on the lower back and alleviate pain.	The review found no strong evidence for using one type of exercise intervention over another when managing patients with CNSLBP.
Heidari et al., 2023 [11]	SR and MA of RCTs	LBP	484	14	Aquatic exercise	+	Mechanisms include the supportive properties of water, which reduce load and stress on the body, potentially allowing for greater movement freedom and reducing pain sensations.	Aquatic workout interventions improve pain intensity, functional disability, and quality of life in adults with chronic LBP.
Yu et al., 2023 [12]	SR and MA of RCTs	CLBP	1108	19	Pilates	+	Pilates may improve pain by enhancing core muscle strength and endurance, promoting better spinal alignment and control, and improving overall somatic stability, which may in turn reduce the biomechanical stresses that contribute to pain.	Pilates is associated with positive pain relief and improvement of functional disorders in patients with chronic low back pain.
Syroyid et al., 2023 [13]	SR and MA of RCTs and non-RCTs	LBP	1661	21	PRT	+	Strengthening the supporting muscles and enhancing spinal stability.	PRT interventions can be beneficial for enhancing physical function in elderly individuals who have general lower back pain (LBP) that is not attributed to lumbar spinal stenosis and who have a body mass index below 27. Furthermore, in older adults with LBP unrelated to lumbar spinal stenosis, PRT interventions have been observed to reduce LBP.
Ram et al., 2023 [14]	SR and MA	CLBP	214	4	High-intensity and low-intensity exercise	No change	Exercise intensity might not significantly influence clinical outcomes in chronic low back pain based on the available data.	There is very low-certainty evidence from a limited number of studies that high-intensity exercise does not provide significant clinical benefits over lower-intensity exercise for reducing disability, improving pain, or enhancing quality of life in people with chronic low back pain.
Prat-Luri et al., 2023 [15]	SR and MA of RCTs	CNSLBP	2391	40	TEPs	+	Increased mobility in these areas may alleviate mechanical stressors contributing to back pain.	Trunk-focused exercise programs had positive effects on pain, disability, quality of life, and trunk performance compared to control groups, and on pain and disability compared to general exercises.
Gilliam et al., 2023 [16]	SR and MA	LBP	558	8	Pilates, yoga, and tai chi	+	Enhancing core strength, flexibility of the lower back muscles, and proper breathing for better posture control.	MB exercise interventions performed by physical therapists are more effective in the short term than non-exercise treatments for low back-related pain and disability, and Pilates interventions are more effective in the long term for pain.
Hernandez-Lucas et al., 2022 [17]	SR and MA of RCTs	NSLBP	1235	4	Strength training, flexibility exercises, and yoga, while the health education component included ergonomics, self-management techniques, pain neuroscience education, and stress reduction techniques	+	Pain reduction is achieved through physical improvements from exercise (such as increased strength and flexibility), combined with cognitive and behavioral changes brought about by educational interventions, which might alter pain perceptions and improve pain coping strategies.	The combination of exercise therapy and health education is more effective than usual medical care in preventing non-specific back pain. This approach not only reduces pain, but also addresses disability and kinesiophobia (fear of movement due to pain), suggesting a comprehensive benefit that extends beyond physical symptoms to include psychological aspects of chronic pain management.
Kazeminia et al., 2023 [18]	SR and MA of RCTs	LBP	926 in total (456 intervention group + 470 control group)	19	Pelvic floor muscle-strengthening exercises	+	Strengthening pelvic floor muscles can provide better support for the pelvic organs and spine, potentially alleviating back pain by stabilizing the lower spine and reducing stress on the back.	Pelvic floor muscle-strengthening exercises significantly reduce low back pain intensity. Therefore, these exercises can be regarded as a part of a low back pain management plan.
Santos et al., 2023 [19]	SR and MA	NSLBP	609	19	MET	+	MET involves using voluntary muscle contractions of the patient against a controlled resistance provided by the therapist, which may help in strengthening muscles, increasing range of motion, and providing pain relief through various physiological mechanisms like improved blood flow and reduced muscle tension.	MET may not be efficient for improving incapacity related to lumbar spine issues, but it can be beneficial in reducing the intensity of LBP.
Belavy et al., 2022 [20]	SR and MA of RCTs	LBP	258,329	6	No physical exercises mentioned, only guideline-adherent surgical referrals for low back pain	No change	N/A	The meta-analysis is out of the scope of the topic, which is about the effect of exercise on low back pain.
Wood et al., 2023 [21]	Secondary analysis of previous RCTs and Comparative MA.	LBP	1897	7	Yoga, Pilates, supervised exercise Programs, McKenzie exercises, home exercises	+	Outcomes matched to exercise treatment targets tend to show greater SMDs.	Matching outcomes to treatment targets in exercise RCTs for CNSLBP might improve the detection of exercise benefits.
Fleckenstein et al., 2022 [22]	SR and MA	CNSLBP	10,084	58	Mixed individualized exercises, sensorimotor training, aerobic exercise, Pilates, McKenzie, back schools, and yoga	+	The effectiveness of individualized exercise may be enhanced when combined with psychological interventions, particularly cognitive-behavioral therapies, indicating that a multimodal approach could be beneficial.	Different exercises may have different effects, and it is important to find a personal set of exercises for each patient.
Shanbehzadeh et al., 2022 [23]	SR and MA	CLBP	763	15	Motor control exercise training	+	Increased muscle activation, particularly of the transversus abdominis.	Increased muscle activation and improved pain and disability levels compared to other interventions, but did not increase resting muscle thickness.
Fernandez-Rodriguez et al., 2022 [24]	SR and NMA of RCTs	CLBP	9710	118	Pilates, mind–body, core-based, strength, stretching, aerobic, McKenzie	+	Increased muscle activation and control, enhanced mindfulness and relaxation, improved core stability and muscle strength.	The study concluded that most types of exercise were beneficial for managing chronic LBP, with Pilates, strength, and core-based exercises being the most effective in reducing pain and disability. Pilates had the highest likelihood for reducing pain and disability based on SUCRA analysis.
Dimitrijevic et al., 2022 [25]	SR and MA	LBP	482	10	Lumbar stabilization exercise, forward head posture-corrective exercises, William training, combining core stability with stretching exercises, sling exercise, corrective exercises of the American National Academy of Sports Medicine, and Pilates exercise	N/A	N/A	This meta-analysis concludes that physical exercises may have a positive impact on the correction of lordosis and hyperlordosis. The results became heterogeneous after the exclusion of one study from the meta-analysis.
Sutanto et al., 2022 [26]	SR and MA	CLBP	2299	47	Isometric and isotonic exercises, motor control exercises	+	Motor control and isotonic trunk training may be effective in reducing low back pain; however, the details are not discussed. However, it was mentioned that isometric trunk training may be effective in reducing the risk of future re-injury based on increased trunk extensor endurance.	The study concludes that isotonic trunk training and motor control are effective in reducing low back pain; however, isometric exercises demonstrated low effectiveness. Further research on how these exercises influence low back pain is needed.
Mapinduzi et al., 2022 [27]	SR and MA of RCTs	NSLBP	1407	12	Motor control exercises	No change	The mechanism remains unclear, but it is suggested that staying physically active may reduce the level of biomechanical change, which may reduce the load on the back, increase the stabilization of joints, and segmental motion.	MSKTs were found to be more effective than MCE in terms of pain reduction, so the combination of both should be used for the most effective outcome.
Kechichian et al., 2022 [28]	SR and MA of RCTs	CLBP	2322	16	Stretching, aerobic, strengthening, and functional tasks, repetitions of step-up, and walking	+	N/A	The meta-analysis suggests that there might be bias in the studies included and concludes that exercises might be effective in reducing pain and improving the functional performance of the participants.
Zhang et al., 2022 [29]	SR and MA of RCTs	LBP	910	18	Tai chi and wu qin xi, aerobic exercise, core stability exercises, resistance training, and integrated training	+	Improved blood circulation, improving joint mobility, improving the protective effect of the spine, reducing stress, and enhancing euphoria.	The meta-analysis concludes that exercises can reduce low back pain in middle-aged and elderly adults. It is recommended to take tai chi and wu qin xi for at least 12 weeks, three times a week.
Arcanjo et al., 2022 [30]	SR and MA of RCTs	CLBP	722	16	PNF	+	Exercises may improve neuromuscular control and motor function by improving movement efficiency, which may reduce pain and improve physical function.	A study suggests that PNF can be used in rehabilitation, since it can improve pain and disability in the lower back. However, the authors suggest that studies and evidence of higher quality are needed to prove this conclusion.
Pocovi et al., 2022 [31]	SR and MA	NSLBP	2362	19	Walking, cycling, and swimming	+	N/A	The authors identified that walking, swimming, and cycling were less effective in reducing low back pain in comparison with other alternatives. So, it is better for patients to try more effective exercises.
Rathnayake et al., 2021 [32]	SR and MA of RCTs	CLBP	1866	9	Self-management interventions with exercise components added	+	N/A	The review identified that SMIs may have a positive effect on patients with CLBP; this effect may vary by duration.
Dal Farra et al., 2022 [33]	SR and MA	NSLBP	521	12	Spinal stabilization exercise, muscle energy technique, Pilates exercise program, whole-body vibration training, Pilates exercises	N/A	Muscle endurance, functional stabilization, proprioception, coordination, and flexibility.	The systematic review and meta-analysis found very low-quality evidence that exercise is effective in improving balance-related outcomes.
Hayden et al., 2021 (a) [34]	SR and MA	CNSLBP	24,486	249	Core strengthening, Pilates, stretching, aerobic exercise	+	Increase in muscle and joint strength, and improvement in muscle function and range of motion.	The study found moderate-certainty evidence that exercise is probably effective for the treatment of chronic low back pain compared to no treatment, usual care, or placebo for pain.
Hayden et al., 2021 (b) [35]	SR and NMA	CNSLBP	20,969	217	Pilates, McKenzie therapy, and functional restoration for chronic low back pain	+	Strengthening of muscles and improvement in posture, increased production of neurotrophic factors, and psychological factors	The study provides strong evidence supporting the effectiveness of Pilates, McKenzie therapy, and functional restoration as exercise treatments for reducing pain and functional limitations in individuals with chronic low back pain.
Quentin et al., 2021 [36]	SR and MA	NSLBP	9588	33	Home-based exercises, trunk, pelvic, leg stretching exercises, and yoga	+	Improvement in pain intensity and functional limitation.	Home-based exercise training was found to be effective in reducing pain intensity and improving functional limitation in patients with non-specific low back pain.
Gao et al., 2022 [37]	SR and MA	CLBP	410	12	Trunk muscle training, PNF, and electromagnetic stimulation	+	Improving trunk proprioception, muscle strength, exercise control, balance, and endurance.	Proprioceptive neuromuscular facilitation showed beneficial effects in relieving pain and improving waist function in patients with chronic low back pain; however, PNF did not significantly improve dynamic balance compared to the control group.
Chen et al., 2021 [38]	SR and MA	LBP	386	8	MFR alone or MFR combined with physical therapy, manual therapy, or exercise therapy for low back pain	No change	Soft tissue release and extension, improvement of local blood circulation, and improvement of muscle pain, stiffness, or excessive fatigue to a certain extent.	The findings suggest that MFR can improve the effect of physical therapy alone and exercise therapy alone, and that MFR can be an effective adjuvant therapy.
Zhang et al., 2021 [39]	MA of RCTs	CNSLBP	1333	18	Motor control exercise and sham treatment, hands-on therapies, and hands-off therapies	+	Improvement in muscle strength and stability, reduction in inflammation, and psychological factors.	Motor control exercise was associated with a significant reduction in pain compared to sham treatment and hands-off therapies for non-specific chronic low back pain.
Sun et al., 2021 [40]	SR and NMA	CNSLBP	7116	31	Low back exercise, targeted muscle strength exercises, and stretching exercises	+	Improvements in muscle strength and flexibility, release of neurotrophic factors, and improved blood circulation.	Low back exercise plus health education is the most effective approach for reducing CNSCLBP in nurses.
Ouellet et al., 2021 [41]	SR and MA of RCTs	NSLBP	1719	18	Tai chi chuan, core stability training, aerobic exercise, resistance exercise, and yoga exercise	No change	Improvement in muscle strength and stability, reduction in inflammation, and oxidative stress.	A study suggests that exercise can lead to pain reduction in musculoskeletal conditions.
Thornton et al., 2021 [42]	SR and MA	LBP	541	14	Exercise, massage, biomechanical modifications, and manual therapy	+	Improvements in muscle strength.	While several treatments for low back pain in athletes improved pain and function, it remains unclear what the most effective treatments are.
Barros-Dos-Santos et al., 2021 [43]	SR and MA of RCTs	CLBP	169 (85 exercise group + 84 control)	4	Physical exercise, interventions with qigong, core stabilization, and aerobic exercises	+	Improved muscle strength and stability, improved blood flow, and tissue healing.	Physical exercise, when performed for at least 6 weeks, is effective in reducing low back pain levels in individuals with chronic LBP.
Mueller and Niederer, 2020 [44]	SR and MR	CNSLBP	2786	50	Stabilization exercises	+	Improvements in muscle strength.	Stabilization exercises had a significant effect on reducing pain and disability in patients with chronic non-specific low back pain. However, there are limitations in exercise trials.
Hanel et al., 2020 [45]	SR and MA	CLBP	2014	17	Exercise training, general practitioner care, and psychological interventions	N/A	Activation of pain inhibitory pathways, improvements in mood, and stress reduction.	Low to very low-quality evidence that exercise training alone may be effective in reducing fear-avoidance beliefs.
Nduwimana et al., 2020 [46]	SR and MA of RCTs	CLBP	3193	31	Walking, yoga, and control interventions	+	The reduction of blood glucose levels in patients with type 2 diabetes maintains functional capabilities, increasing spine motor control and fitness levels, stimulating the brain’s release of serotonin and endorphins, which reduce pain and improve mood.	In the short term, walking has the same effectiveness as control interventions in pain reduction and activity limitation, while yoga is more effective than control interventions. But the results do not provide sufficient proof to support the clinical effectiveness of meditation interventions in CLBP treatment, given the limited number of available trials.
Zhu et al., 2020 [47]	SR and MA of RCTs	CLBP	1466 (exercise only)	18 (9 exercise only)	Yoga, physical therapy, and conventional therapeutic exercises	+	Improving flexibility, mobility, and stability in muscles and joints, spinal alignment and posture, increasing mental and physical relaxation, controlling breath, and improving body awareness.	Yoga was as effective for pain and disability as any other training or physical exercise. Based on the merging outcome, yoga may not increase mental and physical quality level of life. There were no studies studying pain in the long-term.
Domingues-De-Freitas et al., 2020 [48]	SR and MA	CNSLBP	507	5	Pilates exercises	N/A	Improving the effects of kinesiophobia, promoting the movement pattern that the patient is afraid of and that produces pain.	Pilates exercises are more effective at relieving kinesiophobia related to persistent non-specific LBP than limited intervention or no therapy.
Casey et al., 2020 [49]	SR and MA	CLBP	NA	27	Individualized treatment with a cognitive-behavioral therapy component	+	Reducing disability and improving quality of life.	Low-quality proof to propose that multidisciplinary-based rehabilitation is superior to active physical interventions in lowering the pain levels and functional limitations in people suffering from chronic pain over the long and short term.
DeJesus et al., 2020 [50]	SR and MA	LBP	309	5	Specific hip strengthening exercises, mobilization techniques	+	Gluteal muscles provide pelvis stability, which in turn provides a stable base for spine function (especially during single-limb tasks).	For those with LBP, adding targeted hip strengthening exercises to traditional treatment for rehabilitation may help reduce pain and disability.
Amaral et al., 2020 [51]	SR and MA and GRADE recommendations	NSLBP	758	11	Acupuncture, auriculotherapy, exercise (on coping strategies and strength), meditation, self-management, trigger-point acupuncture, and CBT	+	Still unknown.	For elderly patients with low back pain, exercise and trigger point acupuncture have positive short-term effects on pain and impairment. However, information about the long- and medium-term efficacy of conservative therapy is still lacking, namely, regarding other significant outcomes in this particular population.
Wood et al., 2021 [52]	SR and MA	NSLBP	5870	27	Strengthening exercise, spinal stabilization and motor control exercise, stretching focused exercise (yoga, Pilates, tai chi), McKenzie, Godelieve Denys–Struyf or Cesar exercise therapy	+	Spinal stabilization, strengthening muscles, stretching, and flexibility, improving motor control, movement patterns, and activation of muscles.	Training interventions might be more efficient than recent publications, if the results of the therapies match the intervention targets better.
Bernard et al., 2021 [53]	SR of RCTs and MA	NSLBP	200	6	Pelvic floor muscle training (strength, coordination, and endurance exercises)	+	PFM contraction causes the transversus abdominis muscle, which controls the lumbar spine, to co-activate and raise intra-abdominal pressure. Stiffening the sacroiliac joint in women via PFM contraction.	Very low-quality data indicates that there may be little advantage to combining PFMT with another exercise treatment for non-specific low back pain in terms of pain intensity. An integrated lumbopelvic exercise program that includes PFMT with a longer duration is likely to affect pain outcomes in a positive manner, although the change in magnitude did not achieve minimal clinically important change.
Niederer and Mueller, 2020 [54]	SR and MA and MR	CNSLBP	1081	2 CTs + 8 RCTs = 10 studies	Motor control exercise	+	Release of beta-endorphins, both spinal and supraspinal, by activation of μ-opioid receptors.	When comparing exercises focused on stabilization of motor control to other types of control, low- to intermediate-quality proof was found for a lasting favorable impact of the first one on pain and inability in people with LBP. The impacts of the subgroups are less evident, and neither the type, nor the dosage of the comparator, nor the direction of short-term, mid-term, or long-term is made obvious.
Hayden et al., 2020 [55]	MA of RCTs	LBP	3514	27	Exercise therapy (yoga, stretching, etc.), manual therapy, education, or psychological therapy	+	Decreasing pain and improving function.	For persisting non-specific low back pain (LBP) results, exercise therapy had minimal positive effects. It seems that people who are taking pharmaceuticals for their LBP, as well as those who have work that does not demand heavy physical activities, may obtain more advantages from exercising compared to other therapies.
Huang et al., 2020 [56]	SR and NMA of RCTs	LBP	N/A	40	Standard care, normal activity, routinely performed military exercises, ergonomic advice, and video training control	+	N/A	Both training alone and exercise in conjunction with education can stop LBP episodes and absenteeism associated with LBP.
Owen et al., 2020 [57]	NMA	CNSLBP	5578	89 studies	Aerobic, resistance, stabilization/motor control exercise, yoga, Pilates, water-based training, McKenzie exercise	+	Improving mental health, trunk muscle strength, improving physical function.	For adults with CNSCLBP, there is low-quality data to suggest that the most beneficial therapies are Pilates, stabilization control, resistance training, and aerobic exercise training, in expectation of the desired outcome. Additionally, exercise instruction could be more beneficial than direct therapy from a therapist.
Alzahrani et al., 2019 [58]	SR and MA	LBP	95,796	24	LTPA, transportation and domestic activities, walking, cycling, gardening, swimming, aerobics, jogging	+	Increasing muscle strength and flexibility.	Low back pain and physical activity have an inverse relationship. A medium activity level led to a lower prevalence of LBP.
Zhang et al., 2019 (a) [59]	MA of RCTs	LBP	886	11	Traditional Chinese exercises (tai chi, qigong, wuqinxi, yijinjing, baduanjin)	+	Posture control, improvement of core strength, lumbar muscular flexibility, and breathing.	Evidence of a positive effect on lower back pain intensity reduction. Recommend conducting further studies on the topic
Dong et al., 2019 [60]	SR and MA of RCTs	CLBP	680	16	Whole-body vibration exercises	+	Tonic vibration reflexes, spinal and supraspinal neurophysiological mechanisms.	Evidence of a positive effect on chronic musculoskeletal pain. Shows worse results than “traditional treatment.”
Li et al., 2019 [61]	SR and MA	LBP	519	9	Baduanjin	+	Improving back and abdominal muscle strength and spine curvature.	Shows minor improvement in pain relief. Evidence is limited, so a larger-scale and well-designed study is warranted.
Alayat et al., 2019 [62]	SR and MA	LBP	15 (LBP)	1 (LBP)	Stretching, strength, breathing, stabilizing, mobilizing, lumbar isometric and postural exercises, and physical therapy in combination with high-intensity laser therapy	+	Decreasing inflammation, slowing down the transmission of pain signals, and inducing the production of morphine-mimetic substances.	Low-quality evidence of effective pain relief improvement in combination with high-intensity laser therapy.
Alzahrani et al., 2019 [63]	SR and MA of RCTs	LBP	422	3	Walking (Nordic or pedometer-based)	+	Improving overall health, reducing the risk of obesity and musculoskeletal diseases.	Indefinite evidence for no change in lower back pain. Requires further research.
Pourahmadi et al., 2019 [64]	SR and MA	LBP	515	12	Slump stretching, occasionally in combination with stabilization exercises, or exercises	+	Dispersing intraneural edema, restoring pressure gradients, relieving hypoxia, improving associated symptoms in neurogenic pain syndromes, relieving local tension, and reducing inflammation.	Both very low-quality and high-quality evidence for alleviating lower back pain. Recommend researching long-term effects for slump stretching.
Nascimento et al., 2019 [65]	SR and MA	NSLBP	1857	18	Stretching, flexibility, strength, and/or aerobic exercise, yoga, qigong, Pilates, and/or walking	No change	Increasing muscle strength and flexibility.	Limited evidence for a positive change in pain among older adults.
Bernet et al., 2019 [66]	SR and MA	LBP	387	6	Hip strengthening and active resistance exercises, or aquatic-resisted exercises with stretching, strengthening, and aerobic exercises	+	Increasing hip strength and flexibility due to its anatomical interrelationship with the lower back.	No evidence of a statistically significant reduction in pain.
Davenport et al., 2019 [67]	SR and MA	LBP during pregnancy	52,297	32	Aerobic exercises, yoga, specific strengthening exercises, and/or general strengthening exercises	+	Decreasing load on the spine, improving joint stabilization, providing better spinal alignment and segmental motion, and reversing trunk muscle imbalance.	Sufficient evidence for reducing the severity of low back pain. Not possible to distinguish between low back pain subjects and the rest from the paper alone.
Zhang et al., 2019 (b) [68]	SR and MA of RCTs	CLBP	2189 + 2210 (control) = 4399	13	Stretching, postural, respiratory, aerobic, strengthening, cardiovascular, movement, and/or core exercises	+	Promoting a healthy lifestyle and enhancing self-management skills.	Insufficient evidence to support it being universally prescribed. Has a long-term beneficial effect.
Zheng et al., 2018 [69]	MA protocol of RCTs	LBP	N/A	N/A	Whole-body vibration	N/A	Activating muscle fibers, strengthening core stability muscles, relaxing paravertebral muscles, and improving proprioceptive function.	The work provides no useful information, since it is a protocol for a potential study.
Wewege et al., 2018 [70]	SR and MA	CNSLBP	333	6	Progressive aerobic training, or progressive resistance training	+	Improving cardiovascular fitness, strengthening.	Sufficient evidence to show that both methods are effective, while neither is superior. Requires studies that combine the two methods.
Miyamoto et al., 2019 [71]	SR and MA	NSLBP	varies from 80 to 1287	22	General exercise, yoga exercise, stretching, aerobic, strengthening, walking, and manual therapy	+	N/A	For neck pain and subacute and chronic low back pain, exercise therapy is cost-effective, although its results and cost-effectiveness are comparable to those of other therapies.
Luomajoki et al., 2018 [72]	SR and MA	NSLBP	781	11	Movement control exercise, physical exercise, high-load training, specific trunk movements, posture free of pain, physiotherapy, spinal manipulation therapies, muscle energy techniques, therapeutic massage, therapeutic ultrasound, traction	+	Pain duration decreased, pain intensity decreased, posture control improved, strengthening of flexor, extensor, and oblique trunk muscles, and patient education, functional-activity modifications in subject’s trunk movements, individual sensorimotor and cognitive learning.	The MVCE intervention appears to produce better disability improvement than other therapies in individuals with non-specific low back pain and motor control impairments, both in the short and long term. However, the majority of pain relief occurs in the near term, and early patient identification for MVCI is essential.
Lam et al., 2018 [73]	SR and MA	LBP	N/A	17 studies	Physical therapy, manual therapy, joint mobilizations, motion exercise, stretching exercise	+	Pain reduction, improved physical condition.	Moderate to strong evidence suggests that, when it comes to reducing pain and impairment in patients with acute low back pain, MDT CLBP is not superior to alternative rehabilitation options. However, compared to other rehabilitation interventions, there is moderate to strong evidence that MDT is more effective in reducing pain and disability for patients with chronic low back pain; however, the degree of superiority may differ depending on the particular type of intervention being compared to MDT.
Coulter et al., 2018 [74]	SR and MA	CLBP	1176	9	Spinal manipulation therapy, behavioral therapy, exercise therapy, transcutaneous electrical nerve stimulation, interferential currents, low-level laser therapy, yoga, massage, acupuncture, superficial heat therapy, physiotherapy, massage, chiropractic, occupational, and osteopathic therapies	+	Reduction in pain, reduction in disability, improved health-related quality of life.	Both manipulation and mobilization are likely to improve function and reduce pain in people with chronic low back pain, according to moderate-quality data; however, manipulation appears to have a greater overall impact than mobilization. Furthermore, it is thought that both of these medicines are safe, and the application of multimodal programs may also be promising.
Sitthipornvorakul et al., 2018 [75]	MA of RCTs	CLBP	N/A	13	Muscle strengthening, flexibility, and aerobic fitness training, walking	+	Aerobic capacity, body mass index, systolic/diastolic blood pressure, triglyceride levels, and high-density lipoprotein cholesterol levels.	Walking is a simple and easily accessible activity that can be used to minimize disability and relieve pain in the treatment of persistent low back pain, provided that other high-quality research shows different results.
Vanti et al., 2019 [76]	SR and MA of RTs	CLBP	329	5	Walking exercise, walking techniques, circuit training exercise, aerobic exercise, trunk, upper limb, and lower limb strengthening	No change	Pain, disability, quality of life, and fear avoidance improved.	Exercise and walking both have advantages for people with chronic low back pain. It is necessary to conduct more research using bigger sample sizes and various walking techniques.
Shiri et al., 2018 (a) [77]	SR and MA of CTs	LBP	3015	8 RCTs + 6 trials = 14 studies	Stretching exercise, strengthening exercise, aerobic fitness, endurance and coordination exercise, yoga, neuromuscular exercise, combination posture and balance exercise,	+	Improved strength, knowledge about health and work conditions, and health promotion.	According to the meta-analysis, a reasonable guideline for preventing lower back discomfort in the general population is to combine strength training with either stretching or aerobic exercises, performed two to three times per week. Future studies should look into how encouraging spinal exercises affects the amount of time people spend in the hospital and miss work because of lower back discomfort.
Shiri et al., 2018 (b) [78]	MA of RCTs	LBP during pregnancy	2347	11	energy expenditure exercise, water gymnastics, sitting pelvic tilt exercise, strengthening exercises for abdominal, hamstrings, and spinal muscles, low-impact gymnastics and strengthening exercises, aerobic, strengthening, stretching and relaxation, flexibility and endurance, resistance exercises, pelvic floor muscle training, or balance exercises	+	Plausible protective effect from pain, exercise improves muscle strength and endurance, and seems to be more effective in the prevention of new episodes of low back pain.	There is inconclusive information about the effect of exercise on pelvic girdle pain; however, it appears to minimize the incidence of low back pain in pregnant women and the amount of sick leave attributed to lumbopelvic discomfort.
Shi et al., 2018 [79]	SR and MA	LBP	331	8	Aquatic exercise, hydrotherapy, MMPTP + DWR, DWR, and GP	+	Increased physical function.	More research is required to validate the benefits of aquatic exercise for people with lower back pain and improve physical function.
Basson et al., 2017 [80]	SR and MA	NSLBP	1759	40	Neural mobilization exercise, lumbar mobilization exercise, stabilization exercise, slump stretching, manual therapy, nerve gliding exercise, tendon gliding exercises, and splinting	+	Improved functional status scale, median nerve distal motor latency, education in intraneural edema, decreased intraneural edema, decreased temporal summation.	In difficult cases such as persistent lower back pain, neck pain, and plantar heel pain, mobilization techniques help with pain and function and provide direction for carpal tunnel syndrome and pain management.
Shiri and Falah-Hassani, 2017 [81]	SR and MA	LBP	15,475	36	Physical activity	+	Protection from frequent or chronic low back pain.	Physical activity during leisure time may offer a small protective advantage against the development of recurrent or persistent lower back pain. Given the shortcomings of the initial research, it is crucial to interpret these results cautiously.
Geneen et al., 2017 [82]	Cochrane review	CLBP	37,143	381	Resistance and aerobic exercises	+	Improve support around joints, reducing stiffness, and may enhance metabolic exchange in lumbar discs, among other mechanisms.	The study acknowledges the benefits of managed chronic pain through physical exercise, suggesting potential reductions in healthcare use due to improved self-management and reduced pain severity.
Nicolson et al., 2017 [83]	SR and MA	LBP	1045	9	General aerobic exercise, strengthening, flexibility, balance, or body region-specific exercises	N/A	Booster sessions with a physiotherapist to better adhere to therapeutic exercise, motivational strategies, and behavioral graded exercise to improve adherence to exercise.	The meta-analysis offers reasonably reliable evidence supporting the effectiveness of booster sessions with a physiotherapist in enhancing patient adherence to therapeutic exercise among individuals with osteoarthritis. High-quality individual trials indicate that there is emerging evidence to endorse the utilization of patient motivational strategies and graded exercise with a behavioral approach to enhance exercise adherence in individuals with chronic low back pain and osteoarthritis.
Wieland et al., 2013 [84]	SR and MA	CNSLBP	N/A	N/A	Various yoga practices such as physical poses (asanas) and controlled breathing (pranayama), and the incorporation of meditation (dhyana)	+	Improved flexibility and muscular strength, increased mental and physical relaxation, improved body awareness.	The review provides no useful information, since it is a protocol for a potential study.
Coulombe et al., 2017 [85]	SR and MA	CSLBP	414	5	Core stability exercises, general exercises	+	Corset-like stability that leads to a stable Spine and a stable base of support, acts as a transfer point for powerful extremity muscles to generate forceful dynamic contractions reduce the possibility of the injury.	Short-term results showed that core stability exercises were superior to general exercise in reducing pain and enhancing back-specific functional status in individuals with lower back pain.
Gomes-Neto et al., 2017 [86]	SR and MA	LBP	413 stabilization exercises + 297general exercises + 185—manual therapy = 895	11	Stabilization exercises, general exercises, manual therapy	+	Increased muscles strength, enhanced muscle coordination, increased control and coordination of spine and pelvis.	Stabilization exercises were equally effective as manual therapy in reducing pain and disability, and they should be promoted as an integral component of musculoskeletal rehabilitation for individuals with lower back pain.
Moreira-Silva et al., 2016 [87]	SR and MA	NSLBP	N/A	12	Physical activity interventions at the workplace	+	Improve functionality, physical endurance, muscle strength, and joint mobility, reduce localized pain, depression, and social isolation, correct poor posture, increase bone density, relieve stress.	Limited research on the relationship between low back pain and discomfort in the arm, elbow, wrist, hand, or fingers did not yield statistically significant findings. However, there is strong and consistent evidence indicating that interventions involving physical activity in the workplace significantly reduce overall musculoskeletal pain and pain in the neck and shoulders.
Oliveira et al., 2016 [88]	SR and MA	CLBP	N/A	8 published trials + 6 registered trials = 14	Physical activity interventions	N/A	Increasing physical activity levels.	The results indicate that interventions centered around physical activity do not seem to result in a significant change in the objectively measured physical activity levels of individuals with chronic musculoskeletal pain compared to minimal or no intervention. It is important to note that the combined effect observed in the review may alter as more trial results become accessible, considering the numerous registered trials.
Peek and Stevens, 2016 [89]	MA	CLBP	4109	39	All forms of exercises, general exercises	+	Activation of skeletal muscles in a planned and structured manner.	The findings from the meta-analysis indicate that exercise programs for patients which include coordination and stabilization exercises, as well as strength and resistance exercises, have a notable impact on decreasing lower back pain (LBP). However, it is important to note that the study does not offer any supporting evidence for the effectiveness of cardiovascular exercise in reducing LBP.
Yamato et al., 2015 [90]	SR and MA of RCTs	CNSLBP	510	10	Pilates method, several stretching and strengthening exercises	No change	Improvements in strength, range of motion, coordination, balance, muscle symmetry, flexibility, proprioception (awareness of posture), body definition, and general health.	The studies included in the analysis suggest that Pilates is likely more effective than minimal intervention in the short and medium term when it comes to reducing pain and disability and improving function and the overall impression of recovery. However, when it comes to pain and disability, Pilates does not appear to be significantly more effective than other exercise methods in the short and medium term. In terms of function, other exercises were more effective than Pilates in the medium term, but not in the short term.
Searle et al., 2015 [91]	SR and MA of RCTs	CLBP	4462	39	Coordination/stabilization exercises, regular, purposeful, continuous exercise involving major muscle groups, comprised exercise programs with multiple components such as strengthening, stretching, endurance and aerobic training	+	Improve back strength, flexibility, range of motion, and fitness, acute improvement in mood and protection from depression.	The findings indicate that resistance and coordination/stabilization exercise programs are more effective than other interventions in managing chronic low back pain, while cardiorespiratory and combined exercise programs do not appear to provide significant benefits in the treatment of this condition.
Meng and Yue, 2015 [92]	MA of RCTs	CLBP	310	8 clinical studies	Aerobic exercises such as walking, running, treadmill, cycling, and calisthenics	+	Diminish pain intensity and improve physical and psychological functioning.	The meta-analysis offers credible evidence that aerobic exercise can effectively reduce pain intensity and enhance the physical and psychological well-being of individuals with chronic low back pain (CLBP). Therefore, incorporating aerobic exercise into the treatment regimen may be a favorable option for managing CLBP.
Byström et al., 2013 [93]	MA of RCTs	CLBP	N/A	16	Motor control exercises	+	Improved muscle activation and coordination, enhanced core strength, improved posture and body awareness.	Among individuals with chronic and recurring low back pain, MCE appears to outperform various alternative treatments. Nevertheless, further research is required to explore which specific patient subgroups with low back pain are most responsive to MCE.
Bell and Burnett, 2009 [94]	SR	LBP	N/A	10 RCTs + 5 non-RCTs = 15	General exercise such as muscle strengthening, flexibility training or cardiovascular endurance, stabilization exercises	+	Improved core stability, decreased LBP incidence, LBP intensity, and impact of LBP and disability.	While there was robust evidence demonstrating the effectiveness of exercise in alleviating the severity of lower back pain (LBP) and its impact on daily activities, the limited quality of study methods and conflicting findings resulted in only limited support for the use of exercise to prevent LBP episodes in a workplace setting.

Abbreviations: CBT—Cognitive behavioral therapy. CLBP—Chronic low back pain. LBP—Low back pain. MCE/MVCE—Motor control exercises/Motorized or volitional control exercise. MDT—Multidisciplinary treatment. MFR—Myofascial release. NSCLBP—Non-specific chronic low back pain. NSLBP—Non-specific low back pain. PFM—Pelvic floor muscle. PFMT—Pelvic floor muscle training. PNF—Proprioceptive neuromuscular facilitation. SR—Systematic review. MA—Meta-analysis. RCT—Randomized controlled trial. CT—Controlled trial. N/A—Not available or not reported in the study. LTPA—Leisure-time physical activity. TEPs—Therapeutic exercise programs. MET—Muscle energy technique. SMIs—Self-management interventions. SMD—Standardized mean difference. MSKT—musculoskeletal techniques. MVCI—Motorized or volitional control impairments.

**Table 2:** AMSTAR 2.

Author, Year	Q1	Q2	Q3	Q4	Q5	Q6	Q7	Q8	Q9	Q10	Q11	Q12	Q13	Q14	Q15	Q16
Zhang et al., 2023 [7]	+	+	+	Partially yes	+	+	-	+	+	-	+	+	+	+	+	+
Ferraz et al., 2023 [8]	+	Partially yes	+	Partially yes	-	-	-	+	+	-	+	+	+	+	+	+
Gilanyi et al., 2023 [9]	+	+	+	Partially yes	+	+	-	+	+	-	+	+	+	+	+	-
Wong et al., 2023 [10]	+	+	+	Partially yes	-	-	+	+	+	-	+	+	+	+	+	-
Heidari et al., 2023 [11]	+	+	+	Partially yes	-	-	-	+	+	-	+	+	+	+	+	-
Yu et al., 2023 [12]	+	Partially yes	+	Partially yes	+	+	-	+	+	-	+	+	+	+	+	-
Syroyid et al., 2023 [13]	+	Partially yes	+	Partially yes	-	+	-	+	+	-	+	+	+	+	+	+
Ram et al., 2023 [14]	+	Partially yes	-	Partially yes	+	+	-	+	-	-	-	-	-	+	-	-
Prat-Luri et al., 2023 [15]	+	+	+	Partially yes	+	+	+	+	+	-	+	+	+	+	+	+
Gilliam et al., 2023 [16]	+	Partially yes	+	Partially yes	+	+	-	+	+	-	+	+	+	+	-	-
Hernandez-Lucas et al., 2022 [17]	+	+	-	Partially yes	+	+	-	+	+	-	-	-	-	+	-	-
Kazeminia et al., 2023 [18]	+	Partially yes	+	Partially yes	-	-	-	+	+	+	+	+	+	+	-	+
Santos et al., 2022 [19]	+	+	+	Partially yes	+	+	+	+	+	-	+	+	+	+	+	-
Belavy et al., 2022 [20]	+	Partially yes	+	-	+	+	+	+	+	+	+	-	+	+	-	-
Wood et al., 2022 [21]	-	-	+	-	+	+	+	+	-	-	-	-	-	+	-	-
Fleckenstein et al., 2022 [22]	+	+	+	Partially yes	+	+	+	+	+	-	+	+	+	-	+	+
Shanbehzaden et al., 2022 [23]	+	Partially yes	-	Partially yes	+	+	-	-	-	-	+	+	+	+	+	+
Fernandez-Rodriguez et al., 2022 [24]	+	+	+	-	+	+	+	+	+	-	+	+	+	+	+	-
Dimitrijevic et al., 2022 [25]	+	Partially yes	-	Partially yes	+	+	Partially yes	Partially yes	Partially yes	-	+	+	+	+	-	-
Sutanto et al., 2022 [26]	-	-	-	-	+	+	-	+	-	-	+	+	+	+	N/A	+
Mapinduzi et al., 2021 [27]	+	Partially yes	+	-	+	+	+	+	+	-	+	+	+	+	-	+
Kechichian et al., 2022 [28]	-	-	-	-	+	-	-	+	-	-	+	+	+	+	+	+
Zhang et al., 2022 [29]	+	-	+	-	-	-	+	+	-	+	+	+	+	+	-	-
Arcanjo et al., 2020 [30]	-	-	-	-	+	+	-	-	-	-	-	-	-	-	+	-
Pocovi et al., 2022 [31]	+	Partially yes	-	-	+	+	+	+	-	-	-	-	-	+	+	+
Rathnayake et al., 2021 [32]	-	-	+	Partially yes	+	+	+	Partially yes	-	-	-	-	+	-	-	-
Dal Farra et al., 2021 [33]	+	+	+	+	+	-	-	+	Partially yes	-	+	+	+	+	-	+
Hayden et al., 2021 (a) [34]	+	Partially yes	+	Partially yes	+	+	+	+	Partially yes	-	+	+	+	+	+	+
Hayden et al., 2021 (a) [35]	+	Partially yes	+	Partially yes	-	-	-	+	Partially yes	+	+	-	+	+	+	+
Quentin et al., 2021 [36]	+	Partially yes	+	Partially yes	+	+	-	Partially yes	Partially yes	-	+	+	+	+	+	+
Gao et al., 2021 [37]	+	+	+	Partially yes	+	+	+	+	Partially yes	+	+	+	+	+	+	+
Chen et al., 2021 [38]	+	-	+	Partially yes	+	+	Partially yes	+	+	+	+	-	-	+	-	+
Zhang et al., 2021 [39]	+	Partially yes	+	Partially yes	+	+	Partially yes	Partially yes	+	-	+	+	-	+	+	+
Sun et al., 2021 [40]	+	Partially yes	+	Partially yes	+	+	Partially yes	Partially yes	+	-	+	+	+	+	+	+
Ouellet et al., 2021 [41]	+	Partially yes	+	Partially yes	+	+	-	+	-	-	+	+	+	+	-	+
Thornton et al., 2021 [42]	+	Partially yes	+	Partially yes	+	+	Partially yes	Partially yes	Partially yes	-	+	+	+	+	-	+
Barros-Dos-Santos et al., 2021 [43]	+	Partially yes	+	Partially yes	+	+	Partially yes	Partially yes	Partially yes	-	-	+	+	-	+	+
Mueller et al., 2020 [44]	+	Partially yes	+	+	+	+	-	Partially yes	Partially yes	-	-	+	+	-	+	+
Hanel et al., 2020 [45]	+	Partially yes	+	+	+	+	-	Partially yes	Partially yes	-	-	+	+	-	+	+
Nduwimana et al., 2020 [46]	+	+	+	Partially yes	+	+	-	Partially yes	Partially yes	-	-	-	-	-	-	+
Zhu et al., 2020 [47]	+	Partially yes	+	Partially yes	N/A	+	-	Partially yes	+	-	+	+	+	+	+	+
Domingues-de-Freitas 2019 [48]	+	Partially yes	-	Partially yes	+	+	-	+	+	-	+	+	-	+	-	+
Casey et al., 2020 [49]	+	Partially yes	+	Partially yes	+	+	-	Partially yes	+	-	+	+	+	+	+	+
De-Jesus et al., 2020 [50]	+	Partially yes	-	Partially yes	+	+	-	Partially yes	Partially yes	-	-	-	+	-	+	+
Amaral et al., 2020 [51]	+	Partially yes	+	Partially yes	+	+	-	+	Partially yes	-	+	+	+	+	-	+
Wood et al., 2021 [52]	+	Partially yes	+	Partially yes	+	+	-	+	+	-	+	+	+	+	+	+
Bernard et al., 2020 [53]	+	Partially yes	+	Partially yes	+	+	-	+	+	+	-	+	+	-	-	+
Niederer and Muller, 2020 [54]	+	Partially yes	+	Partially yes	+	+	-	Partially yes	Partially yes	-	+	+	+	+	+	+
Hayden et al., 2019 [55]	+	Partially yes	+	-	-	-	-	+	-	-	+	-	+	+	-	+
Huang et al., 2018 [56]	+	Partially yes	+	Partially yes	+	+	-	Partially yes	Partially yes	-	+	+	-	-	-	+
Owen et al., 2019 [57]	+	Partially yes	+	Partially yes	+	+	-	+	+	-	-	+	+	+	-	+
Alzahrani et al., 2020 [58]	+	Partially yes	-	Partially yes	+	+	-	Partially yes	-	-	+	-	+	-	-	+
Zhang et al., 2019 (a) [59]	+	-	-	Partially yes	-	+	-	Partially yes	+	-	+	-	-	+	+	+
Dong et al., 2019 [60]	+	Partially yes	-	Partially yes	+	+	-	+	+	-	+	-	+	+	+	-
Li et al., 2019 [61]	+	+	-	+	+	+	-	Partially yes	+	-	-	+	+	-	+	+
Alayat et al., 2019 [62]	+	+	-	+	+	-	-	+	+	-	+	+	+	+	+	+
Alzahrani et al., 2019 [63]	+	+	-	Partially yes	+	+	-	+	+	-	+	+	+	+	+	+
Pourahmadi et al., 2019 [64]	+	+	-	Partially yes	+	+	-	+	+	-	+	-	+	+	+	+
Nascimento et al., 2019 [65]	+	+	+	Partially yes	+	-	-	+	+	-	+	+	+	+	-	+
Bernet et al., 2019 [66]	+	+	-	Partially yes	+	+	-	+	Partially yes	-	-	+	+	+	-	+
Davenport et al., 2019 [67]	+	+	-	Partially yes	+	-	Partially yes	Partially yes	-	-	+	+	-	+	+	+
Zhang et al., 2019 (b) [68]	+	Partially yes	-	Partially yes	+	+	-	+	Partially yes	-	+	+	+	+	-	+
Zheng et al., 2018 [69]	+	+	-	Partially yes	+	+	N/A	N/A	N/A	N/A	N/A	N/A	N/A	N/A	N/A	+
Wewege et al., 2019 [70]	+	Partially yes	-	Partially yes	+	+	-	Partially yes	Partially yes	-	+	-	-	+	+	+
Miyamoto et al., 2019 [71]	+	+	+	Partially yes	+	+	+	+	+	+	+	+	+	+	+	-
Luomajoki et al., 2018 [72]	+	+	+	Partially yes	+	+	+	+	+	+	+	+	+	+	+	-
Lam et al., 2018 [73]	+	+	+	Partially yes	+	+	-	+	+	-	+	+	+	+	+	+
Coulter et al., 2018 [74]	+	+	+	Partially yes	+	+	-	Partially yes	+	-	+	+	+	+	-	-
Sitthipornvorakul et al., 2018 [75]	+	+	+	+	+	+	+	+	Partially yes	-	+	+	+	+	-	-
Vanti et al., 2019 [76]	+	+	+	+	+	+	+	+	Partially yes	-	+	+	+	+	-	-
Shiri et al., 2018 (a) [77]	+	+	+	+	+	+	+	+	+	-	+	+	+	+	-	+
Shiri et al., 2018 (b) [78]	+	+	+	+	+	+	+	+	Partially yes	-	+	+	+	+	-	-
Shi et al., 2018 [79]	+	+	+	+	+	+	+	+	Partially yes	-	+	+	+	+	-	+
Basson et al., 2017 [80]	+	+	+	+	+	+	+	+	+	-	+	+	+	+	-	-
Shiri and Falah-Hassani, 2017 [81]	+	+	+	+	+	+	+	+	+	-	+	+	+	+	-	+
Geneen et al., 2017 [82]	+	+	+	+	+	+	+	+	Partially yes	-	+	+	+	+	-	-
Nicolson et al., 2017 [83]	+	Partially yes	+	+	+	+	-	-	+	+	+	+	+	-	-	-
Wieland et al., 2013 [84]	+	+	+	+	+	+	+	+	+	+	+	+	+	+	+	+
Coulombe et al., 2017 [85]	+	-	-	-	-	-	-	-	-	-	-	-	-	-	-	-
Gomes-Neto et al., 2017 [86]	+	Partially yes	+	Partially yes	+	+	-	Partially yes	-	-	+	-	-	+	-	+
Moreira-Silva et al., 2016 [87]	+	Partially yes	+	Partially yes	+	-	-	Partially yes	+	+	+	+	-	-	-	+
Oliveira et al., 2016 [88]	+	+	+	Partially yes	+	+	+	+	Partially yes	-	+	+	-	+	-	+
Peek and Stevens, 2016 [89]	+	-	-	Partially yes	-	-	-	-	-	-	+	-	-	-	-	+
Yamato et al., 2015 [90]	+	Partially yes	-	Partially yes	+	+	+	+	+	+	+	-	-	+	-	+
Searle et al., 2015 [91]	+	Partially yes	-	Partially yes	+	-	-	-	-	-	+	-	-	+	-	+
Meng and Yue, 2015 [92]	+	-	-	Partially yes	-	+	-	-	-	-	+	-	-	+	+	+
Byström et al., 2013 [93]	+	-	+	Partially yes	+	-	-	+	-	-	+	-	-	-	-	+
Bell et al., 2009 [94]	+	-	-	Partially yes	-	-	-	Partially yes	-	-	+	-	-	-	-	+

N/A—not applicable.

**Table 3:** Summary of effect sizes and methodological quality of studies comparing exercise types with placebo or other non-exercise interventions.

No	Article	Exercise Type	Effect Size Magnitude	Study Quality
1	Zhang et al., 2023 [7]	Strengthening, Aerobic, Mind–body, Stretching and flexibility	High	Low
2	Ferraz et al., 2023 [8]	Mind–body, Stretching and flexibility	Moderate	Low
3	Gilanyi et al., 2023 [9]	Strengthening, Aerobic, Mind–body, Stretching and flexibility	Small	Low
4	Heidari et al., 2023 [11]	Aquatic	Small	Low
5	Yu et al., 2023 [12]	Mind–body, Motor control, Multimodal, Direction-specific	High	Low
6	Syroyid et al., 2023 [13]	Strengthening	Small	Low
7	Ram et al., 2023 [14]	Multimodal	Not significant	Critically low
8	Prat-Luri et al., 2023 [15]	Trunk-focused exercise	Small	High
9	Gilliam et al., 2023 [16]	Mind–body	High	Critically low
10	Hernandez-Lucas et al., 2022 [17]	Strengthening, Mind–body, Stretching and flexibility	Moderate	Critically low
11	Kazeminia et al., 2023 [18]	Strengthening	High	Critically low
12	Santos et al., 2023 [19]	Muscle energy technique	Small	Moderate
13	Belavy et al., 2022 [20]	Strengthening, Aerobic, Motor control, Stretching and flexibility	Small	Critically low
14	Shanbehzadeh et al., 2022 [23]	Motor control	High	Moderate
15	Dimitrijevic et al., 2022 [25]	Strengthening, Stretching and flexibility	Moderate	Low
16	Kechichian et al., 2022 [28]	Strengthening, Aerobic, Motor control, Stretching and flexibility	Moderate	Critically low
17	Zhang et al., 2022 [29]	Strengthening, Aerobic, Mind–body, Motor control	Small	Critically low
18	Arcanjo et al., 2022 [30]	Neuromuscular exercise	High	Critically low
19	Pocovi et al., 2022 [31]	Aerobic	High	Critically low
20	Rathnayake et al., 2021 [32]	Strengthening, Aerobic, Stretching and flexibility	Small	Critically low
21	Dal Farra et al., 2022 [33]	Mind–body, Motor control	Small	Critically low
22	Hayden et al., 2021 (a) [34]	Strengthening, Aerobic, Mind–body, Stretching and flexibility, Multimodal, Direction-specific	Moderate	High
23	Quentin et al., 2021 [36]	Strengthening, Aerobic, Mind–body, Motor control	High	Low
24	Gao et al., 2022 [37]	Motor control, Neuromuscular exercise	High	High
25	Chen et al., 2021 [38]	Myofascial release	Small	Critically low
26	Zhang et al., 2021 [39]	Motor control	High	Low
27	Barros-Dos-Santos et al., 2021 [43]	Aerobic, Mind–body, Motor control	High	Low
28	Mueller and Niederer, 2020 [44]	Mind–body, Motor control	High	Critically low
29	Hanel et al., 2020 [45]	Strengthening, Aerobic, Mind–body, Motor control, Stretching and flexibility, Multimodal	Small	Critically low
30	Zhu et al., 2020 [47]	Mind–body	High	Low
31	Domingues-De-Freitas et al., 2020 [48]	Mind–body	Small	Critically low
32	De-Jesus et al., 2020 [50]	Strengthening	Small	Critically low
33	Amaral et al., 2020 [51]	Strengthening, Aerobic	Moderate	Critically low
34	Bernard et al., 2021 [53]	Multimodal	Small	Critically low
35	Niederer and Mueller, 2020 [54]	Motor control	Small	Low
36	Hayden et al., 2020 [55]	Strengthening, Aerobic, Mind–body, Stretching and flexibility, Direction-specific	Moderate	Critically low
37	Huang et al., 2020 [56]	Aerobic, Mind–body, Motor control, Stretching and flexibility	Moderate	Critically low
38	Alzahrani et al., 2019 (a) [58]	Aerobic, Multimodal	Small	Critically low
39	Zhang et al., 2019 (a) [59]	Strengthening, Aerobic, Multimodal	Small	Critically low
40	Dong et al., 2019 [60]	Whole-body vibration	Moderate	Low
41	Alayat et al., 2019 [62]	High-intensity laser therapy	High	Low
42	Alzahrani et al., 2019 (b) [63]	Incidental physical activity	Not significant	Low
43	Pourahmadi et al., 2019 [64]	Stretching and flexibility	High	Low
44	Nascimento et al., 2019 [65]	Mind–body	Not significant	Critically low
45	Davenport et al., 2019 [67]	Strengthening, Aerobic, Mind–body	High	Critically low
46	Zhang et al., 2019 (b) [68]	Mind–body	Moderate	Critically low
47	Wewege et al., 2018 [70]	Strengthening, Aerobic	Small	Critically low
48	Miyamoto et al., 2019 [71]	Strengthening, Aerobic, Motor control, Stretching and flexibility, Multimodal	(Cost effectiveness is considered)	High
49	Luomajoki et al., 2018 [72]	Motor control	Small	High
50	Lam et al., 2018 [73]	Direction-specific	Moderate	Low
51	Coulter et al., 2018 [74]	Stretching and flexibility	Small	Critically low
52	Shiri et al., 2018 (a) [77]	Strengthening, Aerobic, Mind–body, Motor control, Stretching and flexibility	Moderate	Low
53	Shiri et al., 2018 (b) [78]	Strengthening, Aerobic, Motor control, Multimodal	Small	Low
54	Shi et al., 2018 [79]	Aquatic	Moderate	Low
55	Basson et al., 2017 [80]	Neural mobilization	Small	Low
56	Shiri and Falah-Hassani, 2017 [81]	Multimodal	Small	Low
57	Geneen et al., 2017 [82]	Strengthening, Aerobic, Mind–body, Stretching and flexibility	No meta-analysis	Low
58	Nicolson et al., 2017 [83]	Strengthening, Aerobic, Motor control	Small	Critically low
59	Wieland et al., 2013 [84]	Mind–body	Small	High
60	Coulombe et al., 2017 [85]	Strengthening, Multimodal	Small	Critically low
61	Moreira-Silva et al., 2016 [87]	Strengthening, Aerobic, Stretching and flexibility	Small	Critically low
62	Oliveira et al., 2016 [88]	Strengthening, Aerobic, Stretching and flexibility	Small	Critically low
63	Yamato et al., 2015 [90]	Mind–body, Motor control	Small	Critically low
64	Searle et al., 2015 [91]	Strengthening, Aerobic, Stretching and flexibility	Small	Critically low
65	Meng and Yue, 2015 [92]	Strengthening, Aerobic	Small	Critically low
66	Byström et al., 2013 [93]	Strengthening, Motor control, Stretching and flexibility, Multimodal	Small	Critically low
67	Bell and Burnett, 2009 [94]	Strengthening, Motor control, Stretching and flexibility	No meta-analysis	Critically low
68	Thornton et al., 2021 [42]	Strengthening, Aerobic, Mind–body	Small	Low
69	Bernet et al., 2019 [66]	Strengthening, Mind–body	Not significant	Critically low
70	Zheng et al., 2018 [69]	Whole-body vibration	Not reported	High
71	Sitthipornvorakul et al., 2018 [75]	Aerobic	Moderate	Low
72	Vanti et al., 2019 [76]	Strengthening, Aerobic, Stretching and flexibility, Multimodal	Not significant	Low
73	Peek and Stevens, 2016 [89]	Strengthening, Mind–body, Motor control	Not reported	Critically low

## Data Availability

Not applicable.
